# Calmodulin Methyltransferase Is Required for Growth, Muscle Strength, Somatosensory Development and Brain Function

**DOI:** 10.1371/journal.pgen.1005388

**Published:** 2015-08-06

**Authors:** Sitvanit Haziza, Roberta Magnani, Dima Lan, Omer Keinan, Ann Saada, Eli Hershkovitz, Nurit Yanay, Yoram Cohen, Yoram Nevo, Robert L. Houtz, Val C. Sheffield, Hava Golan, Ruti Parvari

**Affiliations:** 1 Shraga Segal Department of Microbiology, Immunology and Genetics, Faculty of Health Sciences, Ben-Gurion University of the Negev, Beer Sheva, Israel; 2 Department of Horticulture, University of Kentucky, Lexington, Kentucky, United States of America; 3 Department of Genetic and Metabolic Diseases, Hadassah Hebrew University Medical Center, Jerusalem, Israel; 4 Pediatric Endocrinology & Metabolism Unit, Soroka Medical Center, Beer Sheva, Israel; 5 Pediatric Neuromuscular Laboratory and Pediatric Neurology Unit Hadassah, Hebrew University Medical Center, Jerusalem, Israel; 6 Pesticides and Mycotoxins Division, Aminolab, Weizmann Science Park, Ness Ziona, Israel; 7 Institute of Neurology, Schneider Children's Medical Center of Israel, Petach Tikva, Israel; 8 Department of Pediatrics, Division of Medical Genetics and Hughes Medical Institute, University of Iowa, Iowa City, Iowa, United States of America; 9 Department of Physiology and Cell Biology, Faculty of Health Sciences, Ben-Gurion University of the Negev, Beer-Sheva, Israel; 10 National Institute of Biotechnology in the Negev, Ben Gurion University of the Negev, Beer Sheva, Israel; Max Planck Institute for Molecular Genetics, GERMANY

## Abstract

Calmodulin lysine methyl transferase (CaM KMT) is ubiquitously expressed and highly conserved from plants to vertebrates. CaM is frequently trimethylated at Lys-115, however, the role of CaM methylation in vertebrates has not been studied. *CaM KMT* was found to be homozygously deleted in the 2P21 deletion syndrome that includes 4 genes. These patients present with cystinuria, severe intellectual disabilities, hypotonia, mitochondrial disease and facial dysmorphism. Two siblings with deletion of three of the genes included in the 2P21 deletion syndrome presented with cystinuria, hypotonia, a mild/moderate mental retardation and a respiratory chain complex IV deficiency. To be able to attribute the functional significance of the methylation of CaM in the mouse and the contribution of *CaM KMT* to the clinical presentation of the 2p21deletion patients, we produced a mouse model lacking only *CaM KMT* with deletion borders as in the human 2p21deletion syndrome. No compensatory activity for CaM methylation was found. Impairment of complexes I and IV, and less significantly III, of the mitochondrial respiratory chain was more pronounced in the brain than in muscle. *CaM KMT* is essential for normal body growth and somatosensory development, as well as for the proper functioning of the adult mouse brain. Developmental delay was demonstrated for somatosensory function and for complex behavior, which involved both basal motor function and motivation. The mutant mice also had deficits in motor learning, complex coordination and learning of aversive stimuli. The mouse model contributes to the evaluation of the role of methylated CaM. CaM methylation appears to have a role in growth, muscle strength, somatosensory development and brain function. The current study has clinical implications for human patients. Patients presenting slow growth and muscle weakness that could result from a mitochondrial impairment and mental retardation should be considered for sequence analysis of the *CaM KMT* gene.

## Author Summary

Calmodulin (CaM) is a highly abundant, ubiquitous, small protein, which plays a major role in the transmission of calcium signals to target proteins in eukaryotes. Hundreds of CaM targets are known, and their respective cellular functions include signaling, metabolism, cytoskeletal regulation, and ion channel regulation, to name but a few. CaM is frequently modified after translation, including frequently trimethylation at a single amino acid, however, the role of this methylation is not known. Human patients with a homozygous deletion of the gene that methylates CaM, *CaM-KMT*, are known, but they also have a deletion of additional genes. Thus, to study the role of CaM–KMT, we produced a mouse model in which *CaM-KMT* is the only deleted gene, with the deletion constructed as in the human patients. The model proved to reveal the function of methylation of CaM, since CaM was found to be non-methylated and the methylation of CaM found to be important in growth, muscle strength, somatosensory development and brain function. The current study also has clinical implications for human patients. Patients presenting slow growth and muscle weakness that could result from a mitochondrial impairment and mental retardation should be considered for sequence analysis of the *CaM KMT* gene.

## Introduction

Calmodulin (CaM) is a highly abundant, ubiquitous, small, acidic protein, which plays a major role in the transmission of calcium signals to target proteins in eukaryotes. Hundreds of CaM targets are known, and their respective cellular functions include signaling, metabolism, cytoskeletal regulation, and ion channel regulation, to name but a few. CaM target proteins include, for example, several CaM-dependent protein kinases (CaMK), other enzymes, myosins, receptors, ion channels, and a number of other proteins. The affinity of CaM towards its protein targets varies between the nM and μM ranges [1].

CaM was found to be frequently trimethylated at Lys-115, a solvent-accessible residue (see PDB file 1UP5 5). A limited number of studies have shown that the methylation state of CaM can change in developmental and tissue-dependent manners [2–4], influence the activator properties of CaM with target enzymes [5] and cause phenotypic changes in growth and developmental processes at the level of a whole organism [4,6]. These observations suggest that CaM methylation could be a dynamic mechanism attenuating the interaction of CaM with target proteins influencing a plethora of eukaryotic cellular and developmental processes. [7] However, all observations regarding the role of methylation on CaM were in plants and insects, the effect in vertebrates was to the best of our knowledge not demonstrated. We recently identified the CaM lysine methyl transferase (CaM KMT) and found it to be highly conserved from plants to vertebrates [7]. The human form was previously known as c2orf34 residing at locus 2p21. We reported that deletions of this gene in homozygosity, in the contiguous gene syndrome named the 2P21 deletion syndrome (MIM #606407), are associated with cystinuria, intellectual disabilities, hypotonia, mitochondrial disease and facial dysmorphism [8]. Since the syndrome involves the deletion of 4 genes: *PP2Cβ*, *SLC3A1*, *PREPL* and *CaM KMT* [9–11], the specific contribution of *CaM KMT* to the clinical presentation of the patients could not be determined [12]. Further delineation of the contribution of the deleted genes to the patients' phenotype was revealed by the identification of patients with smaller deletions including only *SLC3A1* and *PREPL*, these patients presented only with hypotonia and cystinuria (HCS) [13–16]. Two additional sibling patients with deletion in *SLC3A1*, *PREPL* and *CaM KMT* presented in addition to the features presented in classical HCS a mild/moderate mental retardation and a respiratory chain complex IV deficiency in one of the patients [17]. Two additional patients with deletion of just *PREPL* and *CaM KMT* presented hypotonia and a mild/moderate mental retardation, the mitochondrial activity was not verified in these patients [18]. Recently it was reported that deletion of *PREPL* causes growth impairment and hypotonia in mice [19], observed also in patients with smaller deletions in the 2p21 interval, not including *CaM KMT* [16]. Thus we set forth to produce a mouse model lacking only the *CaM KMT* gene with deletion borders as in the human 2p21deletion syndrome to be able to attribute the functional significance of the methylation of CaM in the mouse and the contribution of *CaM KMT* to the clinical presentation of the 2p21deletion patients.

## Results

### Generation of mice lacking the coding sequence of the first exon and part of the first intron of CaM KMT

**Fig 1:**
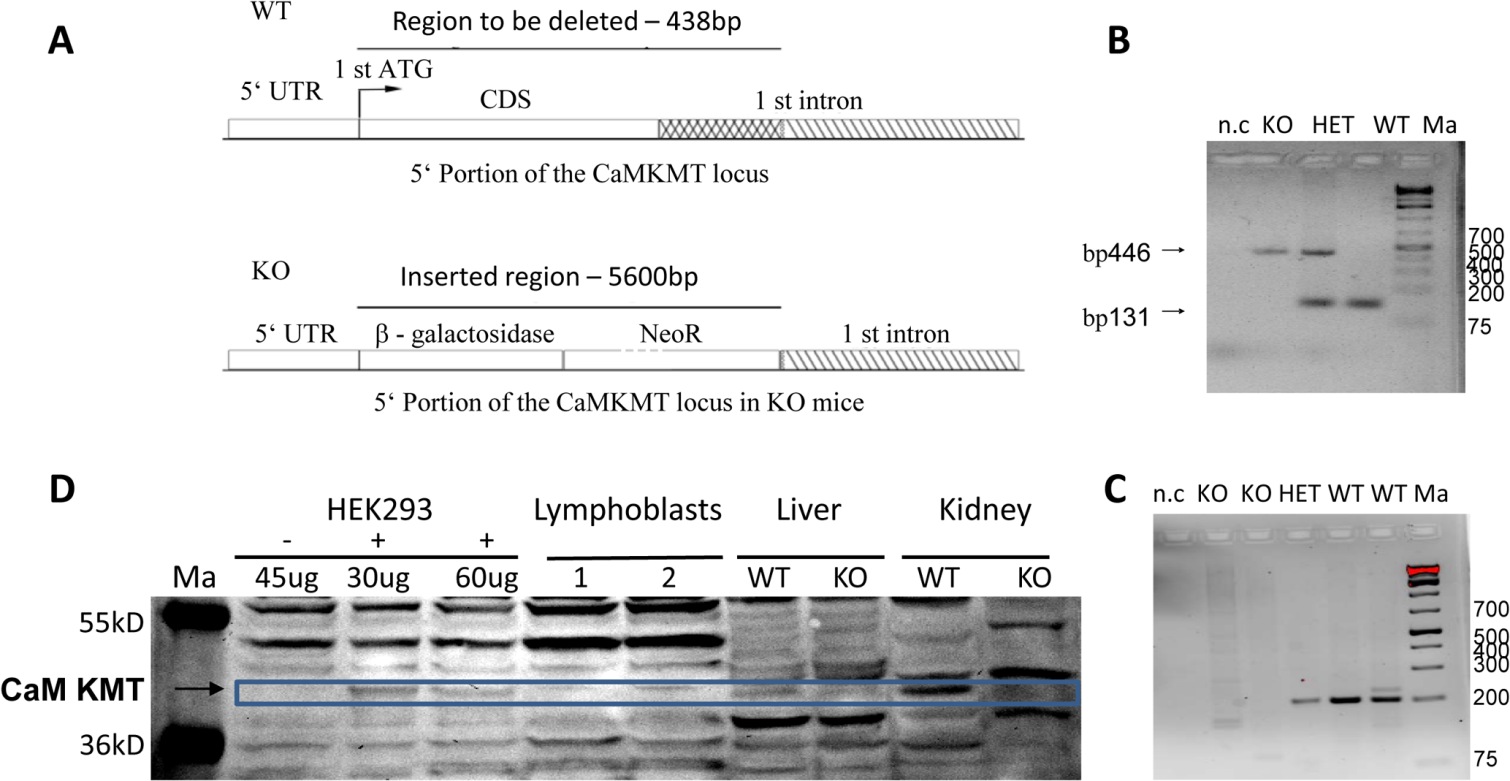
Construct design and validation of the Knock out mice. A. Knock out DNA constructs design. The 138 bp starting with the first ATG of the coding sequence of the gene up to the end of the deletion in patients, 300 bp into the adjacent 1^st^ intron were replaced by a β-gal- Neo cassette of 5600 bp. B. PCR genotyping of the mice. The primers amplifying each of the DNAs are exclusive for that DNA. CaM KMT^-/-^: KO, CaM KMT^+/+^: WT, CaM KMT^+/-^: HET, 1Kb plus DNA ladder (Thermo Scientific): Ma. C. Validation of transcription. RT-PCR on kidney RNA using primers in the 1^st^ and 2^nd^ exons of CaM KMT. CaM KMT^-/-^: KO, CaM KMT^+/+^: WT CaM KMT^+/-^: HET, negative control no DNA: n.c, 1kb plus DNA ladder (ThermoSCIENTIFIC):Ma. D. Validation of translation. Liver and kidney homogenates of CaM KMT^-/-^:-/-, CaM KMT^+/+^: +/+ were analyzed by Western blotting with purified CaM KMT polyclonal antibody [11]. Adjacent lanes on the same gel contained the indicated amounts of lysates of HEK293 transfected (+) or not transfected (-) with myc-CaM KMT pCDNA3 vector to serve as a marker for the size of CaM KMT. Page ruler prestained protein ladder (#SM1811 Fermentas): Ma. 60 μg of human lymphoblasts and mouse tissues and the indicated amounts of HEK293 lysates were separated on 12% PAA gel. The size of the CaM KMT is indicated by the blue box. Although the polyclonal antibodies were affinity purified, the figure represents many cross reactive proteins. The whole Western blot is presented as supplementary S3 Fig.

The mouse homolog of the gene has the same gene structure and spliced forms as the human, and the mouse syntenic region is identical to the human. Thus we expected that by creating a mouse model, in which the coding sequence of the first exon and the adjacent 300 bp of the first intron are deleted, exactly like the border of the deletion of this gene in the 2p21 deletion syndrome patients, will reveal which components of the human phenotype are caused by the specific deletion of CaM KMT. We did not include the 5' untranslated sequence of the first intron in the deletion in order not to affect regulatory regions. The generation of the mouse model began with the design and production of a targeting construct designed to replace the coding sequence and the 5' of the first intron by β -Gal and the Neo gene flanked by floxed repeats. The scheme of the construct is presented in Fig 1A. The results of the PCR reaction used to determine the genotype of the mice is shown in Fig 1B. To ensure that CaM KMT is not expressed in the CaM KMT^-/-^ mice we performed RT-PCR on kidney RNA and demonstrated the absence of a PCR product in the two mice that were verified (Fig 1C, RT-PCR of the CaM KMT^-/-^ with primers for GAPDH that used as control was positive, not shown). We further demonstrated the absence of production of CaM KMT protein in the CaM KMT^-/-^ mice in kidney and liver (Fig 1D). All tissues of the CaM KMT^-/-^ mice appeared normal by histologic examination at 21 days and 9 weeks.

### Expression of CaM KMT

**Fig 2:**
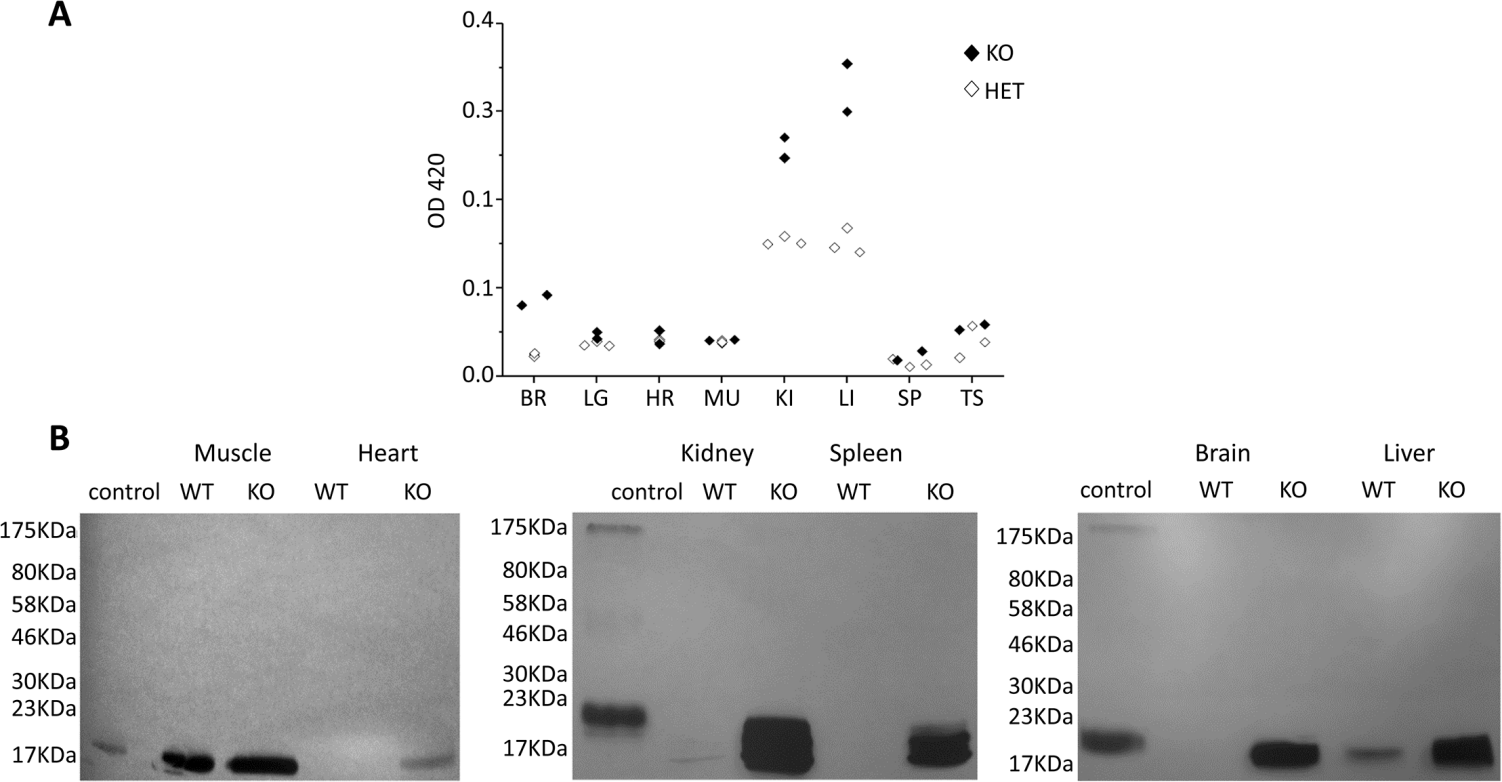
Control of tissue expression of CaM KMT and accumulation of hypomethylated CaM in CaM KMT^-/-^ mice. A. Control of tissue expression. Activity of β-gal of the targeting cassette under the regulation of endogenous regulation was detected by ONPG analysis on homogenates of the detailed tissues. The results from 3 CaM KMT^-/-^ and 2 CaM KMT^+/-^ 3 months old heterozygous male SVJ129 mice are presented. C57Bl6/J mice presented the same pattern. CaM KMT^+/-^ (HET) and CaM KMT^-/-^ (KO) B. Accumulation of hypomethylated CaM. Phosphoimage of lystaes of tissues and organs excised from CaM KMT^-/-^ (KO) or CaM KMT^+/+^ (WT) adult females were incubated with *Hs*CaMKMT and H^3^ Adomet. The control was recombinant CaM incubated with CaM KMT (C). The figure represents one of three experiments with different female mice of both SVJ129 and C57Bl6/J mice. The numbers at the left side of the figures represent the protein size marker.

Since the CaM KMT^-/-^ mouse was constructed with the β-Galactosidase expressed from the initiating AUG of the CaM KMT, the expression of the β-Galactosidase enzyme can be detected by the color production produced with the substrate ONPG (ortho-Nitrophenyl-β-galactoside) [20]. β-Galactosidase activity in different tissues of twelve CaM KMT^-/+^, three CaM KMT^-/-^ and one CaM KMT^+/+^ adult SWJ129 male mice was determined. The highest levels of expression were found in kidney and liver, followed by brain and testis (Fig 2A). Indeed, we succeeded in demonstrating the absence of CaM KMT protein in CaM KMT^-/-^ by Western blotting but only in kidney and liver (Fig 1D, the full blot is presented in S3 Fig), probably because the level of expression of CaM KMT is too low to be detected in other tissues. The deletion does not reduce the transcription of the adjacent gene *PREPL* (S4 Fig). We have also verified whether the deletion affects cystine excretion in urine since patients with the 2P21 syndrome have cystinuria and CaM KMT is highly expressed in kidney. Analysis of cystine in the urine of two CaM KMT^-/-^ C57Bl6J male adult (4 and 10 month old) mice in comparison to comparable CaM KMT^+/+^ indicated no cystine in the urine of the CaM KMT^-/-^ (S5 Fig). Thus, the deletion of CaM KMT does not appear to affect the activity encoded by the *SLC3A1* gene. We have previously demonstrated that in the cells from 2p21 deletion patients the loss of CaM KMT expression resulted in accumulation of hypomethylated CaM compared to normal controls, suggesting that CaM KMT is essential for CaM methylation and there are no compensatory mechanisms for CaM methylation in humans [11]. To evaluate if CaM KMT is essential for calmodulin methylation also in the various tissues of mice we performed an *in vitro* methylation assay using lysates from CaM KMT^-/-^ and CaM KMT^+/+^ controls as a source for CaM as a substrate. The lysates were incubated with purified *Hs*CaM KMT and [^*3*^ *H-methyl*] AdoMet as the methyl donor. A protein of the molecular size of CaM was radioactively labeled in CaM KMT^-/-^ lysates, while this labeling was absent in CaM KMT^+/+^ controls for all tissues tested except for skeletal muscle (and partly in liver) where CaM KMT^+/+^ controls also appeared to be hypomethylated, though to a lesser extent compared to CaM KMT^-/-^ (Fig 2B). The amount of CaM in heart tissues is too low for detection even in the CaM KMT^-/-^ mice. CaM was purified from quadriceps of CaM KMT^+/+^ adult females and analyzed by MS/MS for the presence of methylation. Depending on the experiment, both forms of CaM, methylated and not methylated were detected in the samples (S6 Fig).

**Fig 3:**
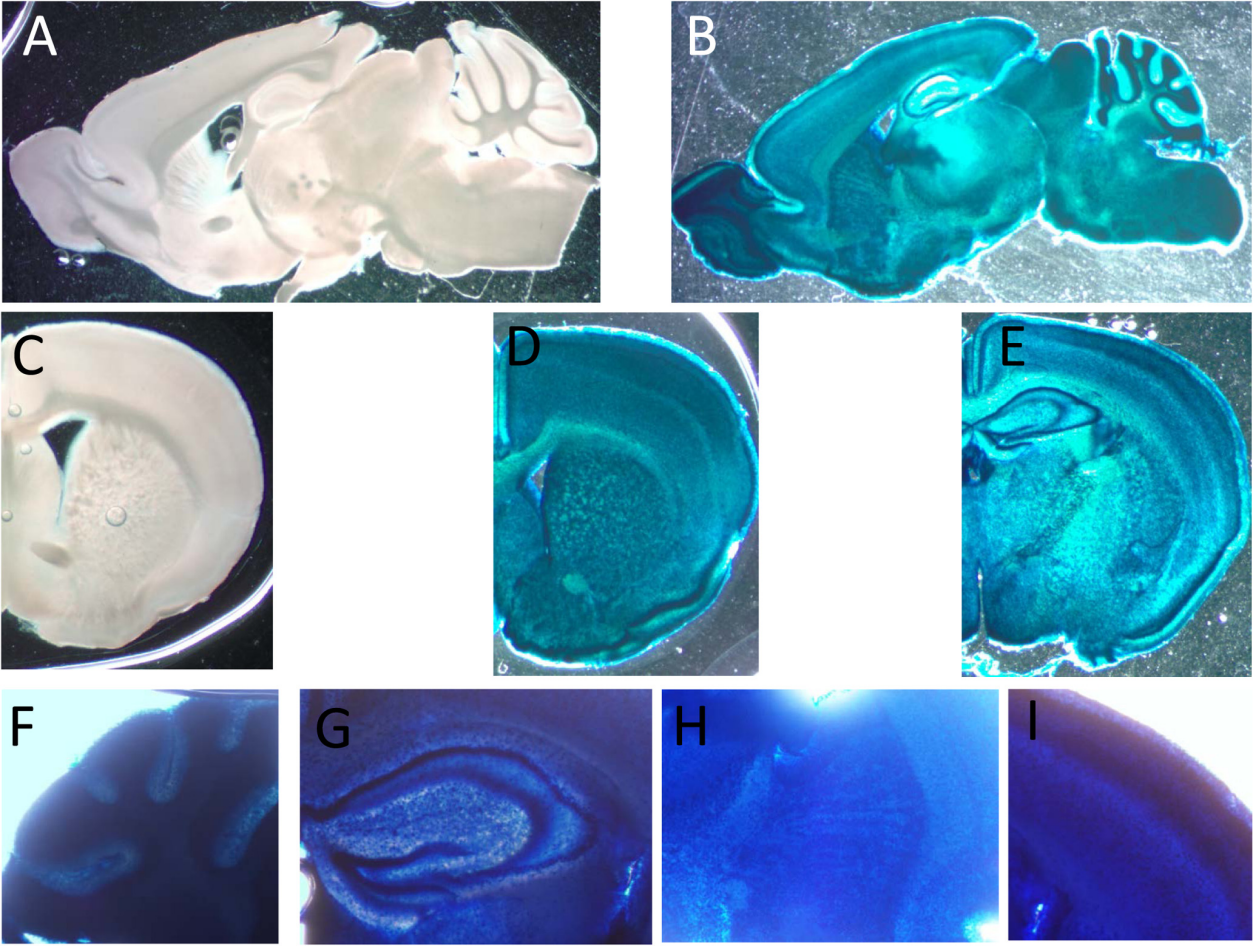
Expression of CaM KMT in the brain of adult C57Bl mice. Representative staining for β-Gal activity sagittal (B) and coronal (D and E) sections of CaM KMT-/- mice brains, compared to CaM KMT+/+ (A and C). The figure represents results of 4 different adult male C57Bl6/J mice brains. Magnified images of cerebellum lobules (F), hippocampus (G), striatum (H) and M1 region of the cerebral cortex (I) Images exemplify the expression in all cells in all the brain regions. The nuclear staining is attributed to the nuclear localization signal in the β galoctosidase gene.

The pattern of expression of CaM KMT was also verified in brain by β-Gal staining (Fig 3). It was found to be ubiquitously expressed and mostly homogenous in all brain tissue Fig 3B and 3D and 3E. Examples of the expression in the cerebral cortex, striatum and cerebellum are presented in magnified photographs (Fig 3F–3I). This pattern of distribution is in agreement with the expression of CaM KMT as shown in the Allen Brain Atlas project (http://www.brain-map.org/).

### Verification of mitochondrial defect

**Fig 4:**
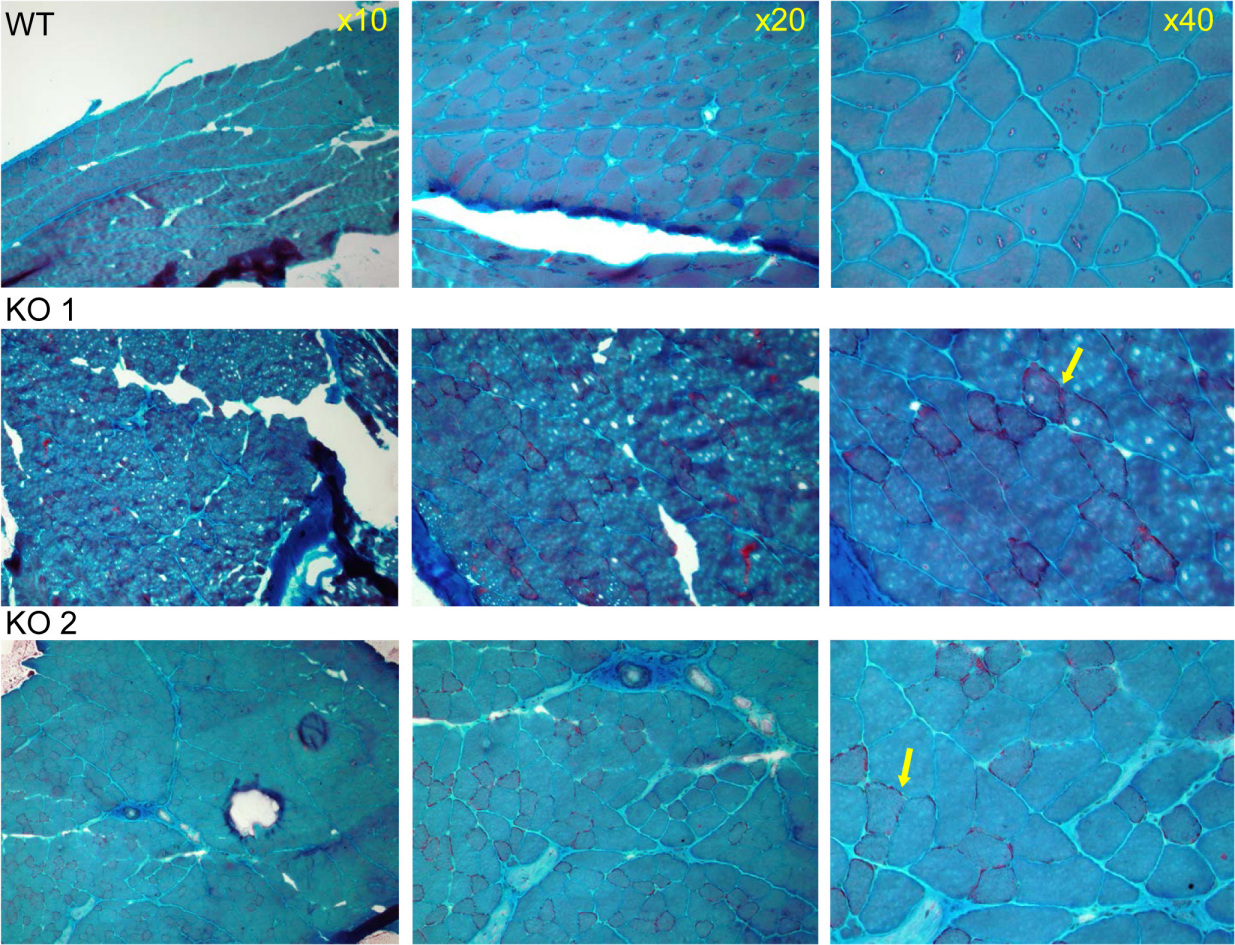
Muscle pathology of CaM KMT-/- mice demonstrated by Gomori-Trichrome staining. The myopathic feature is shown by the variation in fiber size of the two CaM KMT^-/-^ samples: KO (middle and lower panels), not observed in the sample of the comparable CaM KMT^+/+^ WT (upper panel). The yellow arrows point the ragged red fibers in the X40 magnification.

Since the patients of the 2P21 deletion syndrome demonstrated mitochondrial defect in muscle biopsies, including ragged red fibers [8] we verified possible mitochondrial functional deficiency by Gomori-Trichrome staining of the quadricep muscles of two CaM KMT^-/-^ C57Bl6J male adult (8 month old) mice in comparison to comparable CaM KMT^+/+^. Muscle pathology was consistent with variation in fiber size (myopathic feature), also observed in patients with deletions of *SLC3A1*, *PREPL* and *CaM KMT* (17) and occasionally peripheral sub- sarcolemmal accumulation of mitochondria reminiscent of ragged red fibers (Fig 4).

**Fig 5:**
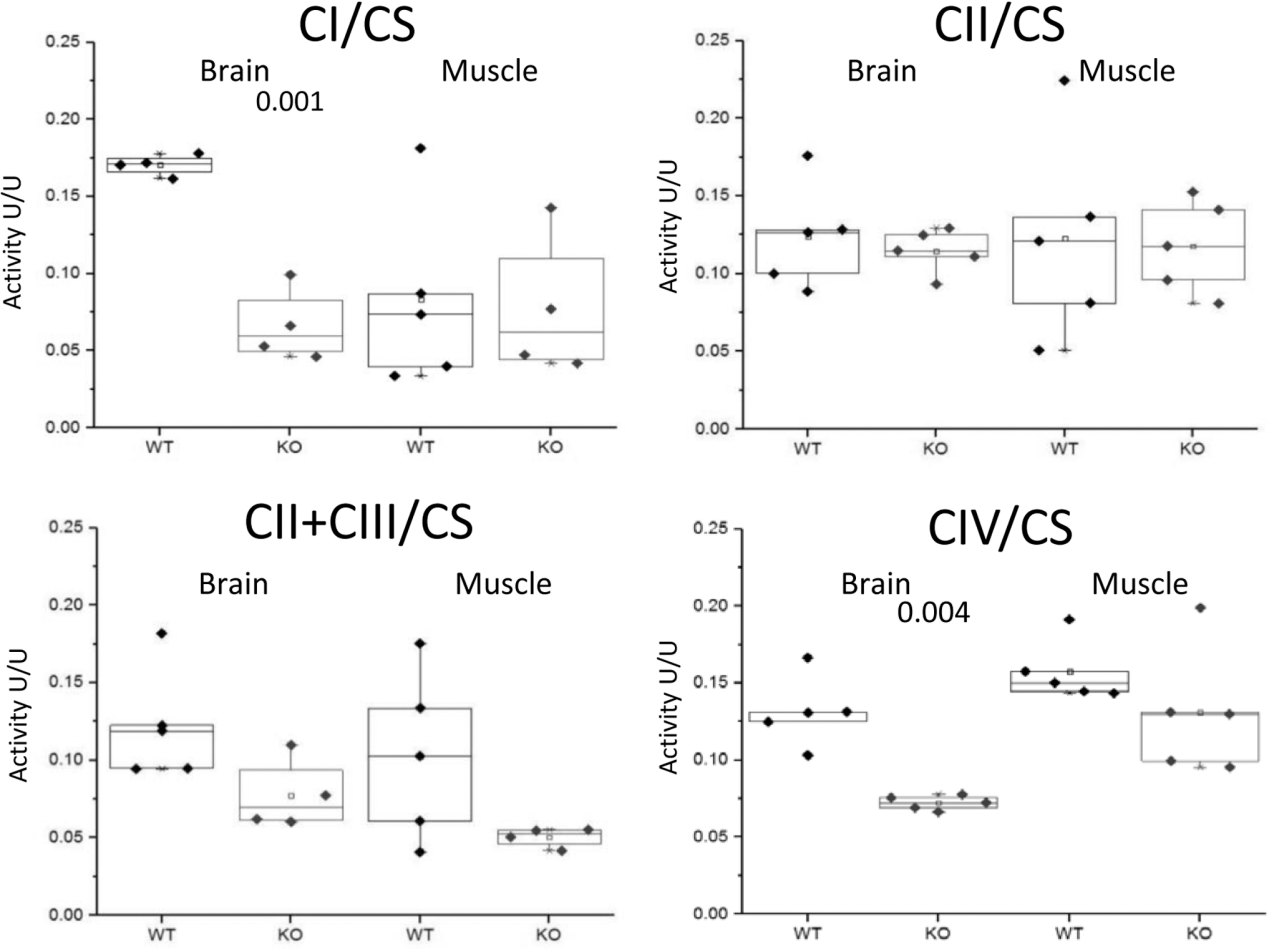
Enzymatic activities of respiratory chain complexes. The enzymatic activities of respiratory chain complexes I,II,II+III and IV were measured by spectrophotometric methods in mitochondria isolated from CaM KMT^+/+^ (WT) and CaM KMT^-/-^ (KO) brain and skeletal (hind limb) muscle. Results are presented as ratio (U/U) to citrate synthase (CS). Each point represents an experiment duplicate performed in different occasions. Significant difference between WT and KO for each is presented above graph (2 tails-student T-Test). For complexes II+III in brain and muscle p values were indicative (0.056 and 0.097, respectively).

Since the human P21 deletion syndrome also had reduced respiratory chain deficiency of all complexes except complex II [8] and the patients with SLC3A1, PREPL and CaM KMT showed reduced respiratory chain deficiency of complex IV [17] we further analyzed the enzymatic activity of the respiratory chain complexes in brains and muscles of the CaM KMT^-/-^ C57Bl6J five adult (8 month old) mice in comparison to comparable CaM KMT^+/+^ and observed a marked decrease of both complex I and IV activities in the brain (Fig 5). This defect was only partially reflected in the muscle as complex I was unaltered and complex IV activity was moderately but not significantly decreased. The combined activities of II+III were decreased in both tissues. Complex II, which is solely nuclear encoded, was not affected in either tissue in agreement with the findings in the 2P21 deletion syndrome patients [8].

### Sensory and motor development

All developmental and behavioral analyses at all ages were performed on mice that were backcrossed for 10 generations into the C57Bl6J strain and comparisons done between siblings of heterozygous parents. The effect of CaM KMT on the development of sensory and motor reflexes was addressed during the first month of mice life. During this period mice were daily weighed as control for gross growth.

**Fig 6:**
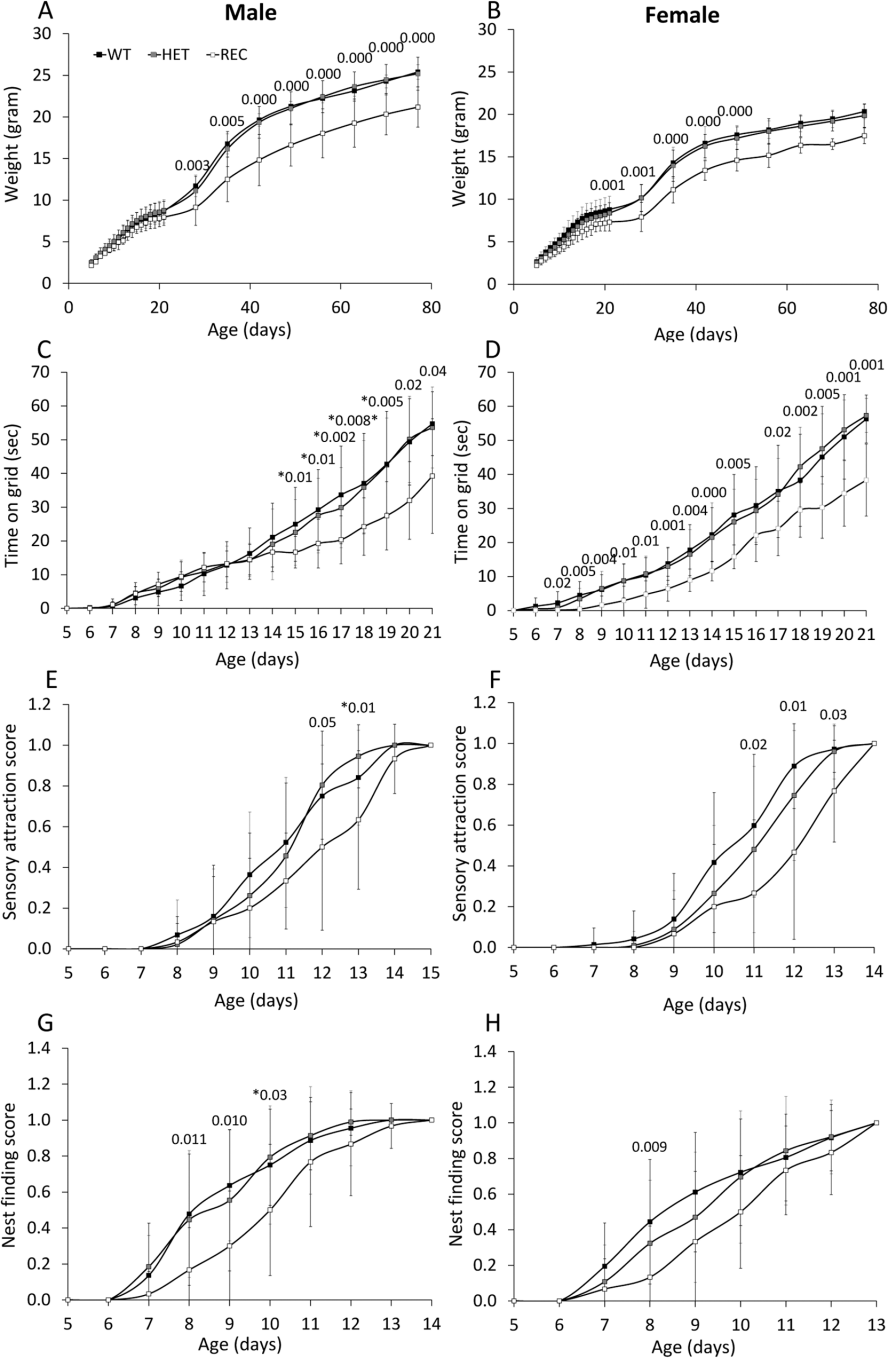
Morphogenic development and sensory–motor reflex development of newborn mice. Developmental milestones were analysed in CaM KMT^+/+^ (WT), CaM KMT^-/+^ (HET) and CaM KMT^-/-^ (KO) mice. Newborn weight of male (A) and of female (B). Development of the ability to cling to a vertical wire is presented as the time held on the wire by male and female (C and D, respectively). Newborn mice responsiveness to sensory attraction (E and F). Nest finding score (G and H). Sensory attraction and nest finding scores are represented on a scale from 0 (no response) to 1 (full response in 3 trials). Mean ± SD, n = 7–15 mice in each group. Significant difference between WT and KO is presented above graph (ANOVA post-hoc). For the gaining of weight in parts A and B most of the differences had a p value of 0.000, the less significant one was 0.029. These could not be presented above the graph. Values presented with * were between the KO and HET, due to a small number of the WT that didn't give significance. N = 15–32 (the exact number of mice in each group and test is shown in S1 Table).

Reduced weight was obtained in CaM KMT^-/-^ newborns of both sex compared to CaM KMT^+/+^, while the CaM KMT^+/-^ mice gained weight similar to the CaM KMT^+/+^ mice. Male CaM KMT^-/-^ mice weight was in average 4gr lower compared to CaM KMT^+/+^ (P<0.001) and 2.5 gr lower in CaM KMT^-/-^ female, compared to CaM KMT^+/+^ female mice (P<0.003). These differences were preserved until the age of 80 days as indicated in Fig 6A and 6B. Muscle strength was tested by the time newborns were able to hold to a vertical wire as described in the methods section. Male mice of all groups performed similarly in the first couple of weeks, while at P17 the CaM KMT^-/-^ mice were capable to hold to the wire for significantly shorter time compare to the CaM KMT^+/+^ and CaM KMT^+/-^ groups. In female mice a similar effect was evident already at age of 7 days. In both sexes this difference was still evident at age of 21 days when the CaM KMT^-/-^ mice could hang to the wire for 13sec less than CaM KMT^+/+^ (Fig 6C and 6D and S1 and S2 Tables). ANOVA for repeated measurements in female: F_2,64_ = 13.05, P<0.001 and male F_2,48_ = 5.6 p<0.001. Mice attraction to auditory stimulus was observed in control mice already at day 8–9 and became prominent in most CaM KMT ^+/+^ mice by the age of 11–12 days. The CaM KMT^-/-^ mice of both sex were delayed to acquiring this function compared to the CaM KMT^+/+^ (ANOVA, F_2,81_ = 10.24, P<0.001 and F_2,100_ = 7.56, p<0.01, in male and female, respectively), however by the age of 15 days in male and 14 days in female all mice fully reacted to the auditory stimulus, as shown in Fig 6E and 6F and in S1 and S2 Tables. Nest finding testing depends on mice sensory identification of nest location, their motor ability and motivation to return to the cage. Mice of the CaM KMT^-/-^ show reduced scores in the nest finding test compared to CaM KMT^+/+^ and CaM KMT^+/-^ groups during days 8–10, (ANOVA, F_2,64_ = 6.35, P<0.003; F_2,48_ = 4.2 p<0.01), in male and female, respectively, while at later ages all groups reached maximal scores (Fig 6G and 6H). A summary of developmental evaluation statistical values can be seen in S1 Table: Developmental profile test and S2 Table: Developmental profile test statistics.

### Behavior of the adult mice

**Fig 7:**
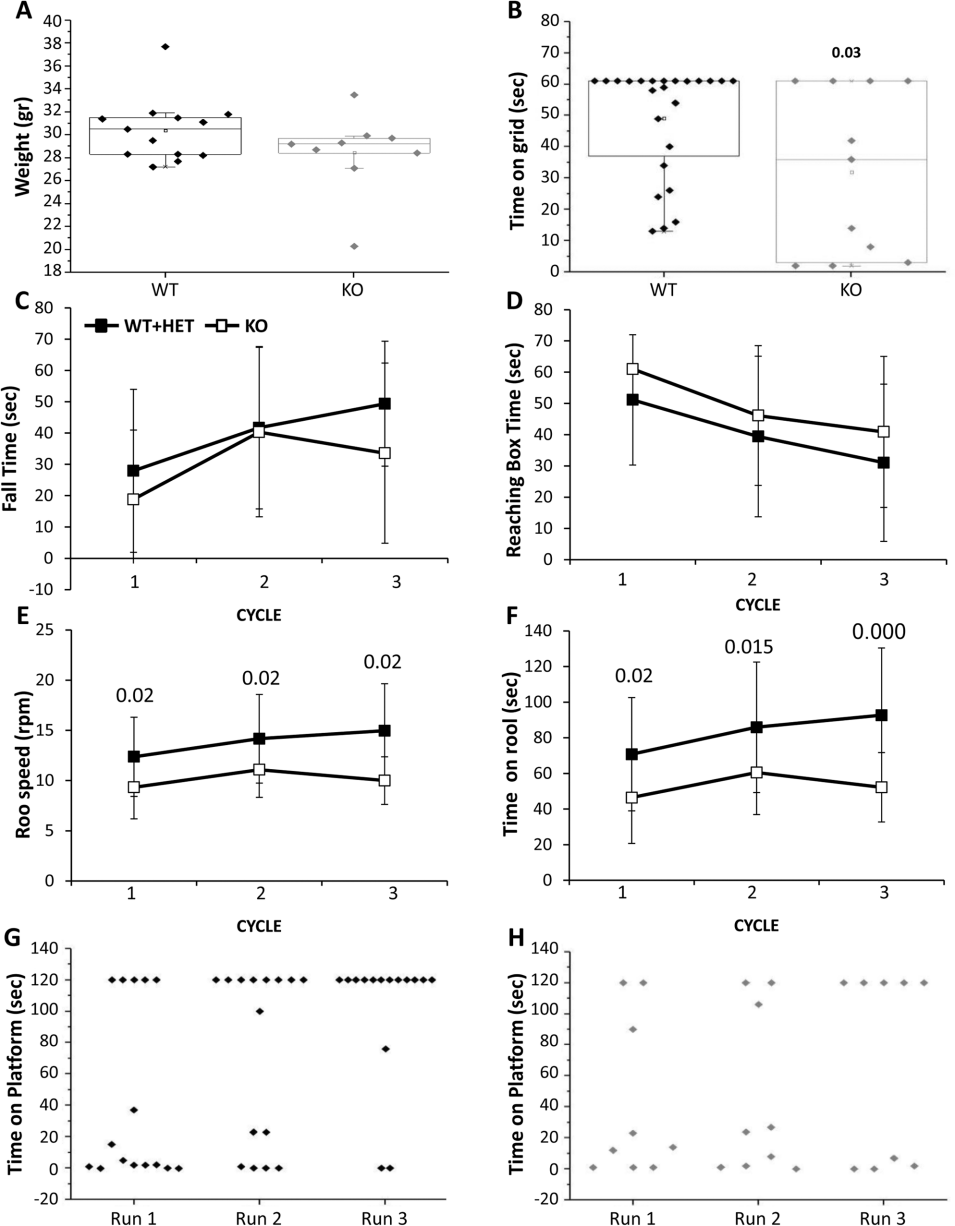
Adult mouse behavior. Male mice tested were older than 3 months. A. Mice weight. B. The duration of time the mice held to horizontal metal wire grid is shown for each individual mouse. The significant p value is presented (ANOVA post-hoc). The balanced beam was tested in 3 consecutive trials: The time on the beam (C) and the time to reach escape box (D). Mice performance in the rota-rod test in 3 trials: The time the mice stayed on the rotarod (E) and the maximal speed in which the mice successfully stay on the rotarod (F). The significant p values are presented (ANOVA post-hoc). The time mice spent on vibrating platform is shown for WT (G) and KO (H), maximal time on platform was 120 sec. Mean ± SD are presented for C-F, For the balance beam and Rotarod tests: twelve mice for each genotype. CaM KMT^+/+^: WT, CaM KMT^+/-^: HET, CaM KMT^-/-^: KO.

Characterization of mouse behavior was performed only in males older than 3 months. Due to similar performance of the CaM KMT^+/+^ and CaM KMT^+/-^ mice the results of these groups were included in one group. Male weight among the mice that survived to this age did not differ significantly between groups (Fig 7A). Tail length was also measured as an additional index for body growth and showed similar values between the CaM KMT^+/+^ and CaM KMT^-/-^ groups (7.3 +/-0.1 cm and 7.1 +/- 0.1 cm, respectively; Student t-test, t = 1.03, p = 0.3). All mice were first tested in the open field, general exploration evaluated by the distance walked. Walking velocity and mobility showed no difference between genotypes. No significant differences between groups were observed when the time in the center of the arena was analyzed as an index for mice anxiety. The majority of CaM KMT^-/-^ mice were not able to cling to a grid for the duration of the test and held significantly shorter time compared to the mice of the CaM KMT^+/+^ group where most of the mice griped to the grid 60 sec, the maximal time tested (49.2 ±17.6 and 29.5±25.5, in CaM KMT^+/+^ and CaM KMT^-/-^, respectively, Student t-test, t = 2.3, p<0.03) as shown in Fig 7B. Motor performance and coordination was addressed on the balance beam by the analysis of time to fall from the beam and the time required reaching one of the two escape boxes located at the end of the beam. Mice of both groups improved their performance in the repeated trials and stayed longer on the beam compared to previous trial (time to fall, F_2,19_ = 6.7, p<0.01 and F_2,10_ = 2.7, p<0.05; in CaM KMT ^+/+^ and CaM KMT^-/-^, respectively, ANOVA for repeated measurements) (Fig 7C), however only the CaM KMT^+/+^ group improved the time to reach the box (time to box F_2,22_ = 7.9, p<0.01 and F_2,7_ = 2.7, p<0.1 in CaM KMT^+/+^ and CaM KMT^-/-^, respectively) as depicted in 7D. Higher ability of motor function and coordination is required to hold to an accelerating rotating rod, when rotation speed is gradually elevated. Shorter time on the rotating rod and lower speed were characteristic of CaM KMT^-/-^ mice, compared to the CaM KMT^+/+^ mice performance (F_1,35_ = 9.5, p<0.01 and F_1,35_ = 9.4, p<0.01, for duration and speed, respectively). In addition, while the CaM KMT^+/+^ mice improved their performance during the 3 cycles of the test (F_1,22_ = 6.8, p<0.01 and F_1,35_ = 6.1, p<0.01, for duration and speed, respectively, ANOVA for repeated measurements), the CaM KMT^-/-^ mice did not improve during the training and maintained similar scores through the test. Finally, avoidance memory was tested by the passive avoidance test. Mice were trained 10 trials per day in the passive avoidance apparatus to avoid stepping down from the platform. The duration until mice placed 2 limbs on the chamber floor was recorded as an index for learning. The score at the last trial of each training day is presented for each individual mouse in Fig 7G. Larger proportion of mice of the CaM KMT^+/+^ group improve their score within the training days and did not step down from the platform at the last trial of the test; the average time on the platform in the CaM KMT^+/+^ and CaM KMT^+/-^ group was 44.3±56.2 sec in the first day and 101.1±42.6 in the last day of training, F_1,13_ = 6.9, p<0.01, while the mice of the CaM KMT^-/-^ groups failed to acquire learning of the task; mice of this group acquired an average of 42.4±51.9 sec on the platform in the first day and 67.7±62.1 at the last day of training, F_1,7_ = 1.37, p<0.3 where about a half of the mice step down from the platform (Fig 7H). It should be noted that the performance at the first day of the test was similar in both groups, thus the differences obtained in the following days may be due differences in the learning process. In addition to exclude the possibility that the differences obtained in the passive avoidance test were due to genotype dependent differences in the somatosensory response mice were tested in the hot plate test. Similar response time in the hot plate test, characterized by 12.43 +/- 1.0 sec; 13.04+/-0.8 sec in the CaM KMT^-/-^ and the CaM KMT^+/+^ mice was obtained. A summary of the statistical analysis of adult mice behavior is presented in S3 Table. Adult mice behavior statistics.

To verify whether the reduction in muscle strength is accompanied by changes in the muscle structures we performed Hematoxilin and Eosin staining on triceps brachii (fore limb) and quadriceps (hind limb) muscles from CaM KMT^+/+^ adult mice. No abnormalities were revealed.

Overall, developmental delay was observed in mice carrying the CaM KMT^-/-^ genotype compared to CaM KMT^+/+^ mice, while the heterozygote mice development was comparable to that of the CaM KMT^+/+^ newborns. Although some of the criteria were acquired with age, impaired motor function, limited motor learning and failure to learn the avoidance of aversive stimulus characterized the CaM KMT^-/-^ mice at adulthood. Heterozygote CaM KMT^+/-^ behave similarly to the CaM KMT^+/+^ mice in all tested tasks, indicating that one copy of the gene is sufficient to support neurodevelopment.

## Discussion

Based on interest in the importance of methylation of CaM and the underlying causes of the 2P21 deletion syndrome, we have generated a mouse model lacking only the CaM KMT gene with the borders of the deletion as in the human syndrome.

Here we show for the first time that the protein CaM KMT is essential for normal body growth and somatosensory development as well as for muscle strength and the proper functioning of the adult mouse brain. Developmental delay was demonstrated for somatosensory function (attraction to sensory stimulus) and muscle strength (clinging on a wire) and for more complex behavior which involved both basal motor function and motivation (nest finding). We exclude the possibility that newborns did not find the nest due to impaired smell sensation since these mice were similar to the CaM KMT^+/+^ in the odor test. Developmental delay was observed at the young age for the tasks involving somatosensory processing, namely the sensory attraction and nest finding. In contrast, the task that involves mostly motor function, namely hanging on a wire, lower performance of the CaM KMT^-/-^ relative to the CaM KMT^+/+^ persists to adulthood. At adulthood CaM KMT^-/-^ perform similarly to the CaM KMT^+/+^ mice in low demanding tasks such as exploration in the open field and balanced beam. However, in tasks which require higher levels of motor and coordination function, namely rota-rod, the CaM KMT^-/-^ did not improve their performance with training and performed below the CaM KMT^+/+^ mice. Notably, by repeated training on the rota-rod test CaM KMT^+/+^ improve their performance, as reflected both by the time they stay on the apparatus and the speed they manage to adjust to (Fig 7E and 7F). This change in time, which is indicative of motor learning was not seen in the CaM KMT^-/-^ mice. The complex coordination required for this task involved the action of cerebellum and other components of the motor control system [21,22], but also involve the function and plasticity of the neuromuscular junction [23] and muscular strength. Transmission on the neuromuscular junction was not tested in the current study; however muscle myopathy was observed by the Gomori Trichome staining of in the striated muscle and may suggest that lower function in the rota-rod is of peripheral source.

The expression of CaM KMT β-gal obtained in cells of all brain structures suggests that this protein is required for the function of all cells in the central nervous system. If indeed required for the proper function of all neurons we would expect a gross behavioral phenotype. However it is possible that CaM methylation is essential for the execution of particular tasks, as presented in the results, for example the abundance of CaM KMT distribution in all cells of the cerebellum and low functioning in the rota-rod test suggests that CaM lysine modification is essential for complex motor coordination and motor learning. An additional aspect of learning, where the CaM KMT^-/-^ mice failed to improve with training, was shown in the passive avoidance test involving learning of an aversive stimulus. It is possible that CaM methylation at one or more of the brain regions supporting this behavior, in particular the amygdale and cortico–hippocampal network, is essential for this type of learning [24] and memory consolidation [25,26].

In a recent evaluation of three older 2P21 syndrome patients, 20–30 years of age, it was found that, although able to walk and eat they are entirely dependent on their parents. They demonstrated severe mental incapacity and lack of speech despite normal hearing. This presentation of the patients, which could be more severe than that observed in the mouse model, may represent the effect of the additional missing gene the phosphatase *PP2Cβ* **(** *PP2Cβ*) for which no mouse model is yet reported.

The impairment of complexes I, III and IV of the mitochondrial respiratory chain in brain was anticipated based on the 2P21 deletion patients. Surprisingly the muscle complexes were less affected, indicating possible tissue variability. This could possibly be due to factors with overlapping functions present in muscle but not in brain. Notably complexes I, III and IV are dependent on mitochondrial DNA encoded subunits while complex II, the only one with unaffected activity is synthesized from nuclear DNA. Thus in accord with the previous finding in patients we hypothesize that CaM KMT could possibly be involved in the production of proteins encoded by mitochondrial DNA genes.

The present mouse model contributes to the evaluation of the role of methylated CaM, since in the tissues where CaM is methylated the elimination of the CaM KMT activity left the CaM non-methylated (Fig 2B), indicating there is no compensatory mechanism and CaM KMT is the only enzyme that methylates CaM. This is in agreement with our previous finding in lymphoblastoid cells derived from the 2P21 deletion patients [11]. The finding of hypomethylation of CaM in muscle was confirmed by mass spectrometry analysis performed on CaM extracted from the adult female control mice. Analyses by MS/MS lack the ability to be quantitative and consequently it wasn`t possible to estimate the amount of CaM that was not methylated with this experiment. One previous article indicated that 68% of skeletal muscle CaM is not methylated in adult male rats (it is also reported that 12% of liver CaM is not methylated) [27]. Heart CaM is also indicated to be undermethylated but we didn`t observe the same results in our samples. An older article [28] seems to have indication of undermethylated muscle CaM in rabbit. The hypomethylation of CaM in muscle and the major effect observed in muscle is presently not understood and requires further study. It is possible that the methylation may be needed at a specific window during muscle development and is not necessary at the time it was tested in our study, which was adult mice. Indeed the muscle phenotype is observed already in the development of the newborn mice with the reduced ability of the CaM KMT^-/-^ mice to hold on the wire. An additional possibility is that the muscle phenotype could be caused by a compromise in brain function. Indeed, the tasks requiring brain involvement were affected. These findings can also point to the importance of CaM KMT in proper brain function. Methylation of CaM was reported in six regions tested: caudate nucleus, cerebral cortex, superior colliculus, brain stem, diencephalon and pituitary gland [29]. Thus, the impairment in intellectual ability observed in human with deletion of CaM KMT with additional genes [8,17,18] could be attributed to CaM KMT.

The present study enables to better assess the contribution CaM KMT to the clinical presentations of patients with different deletions involving it and additional adjacent genes homozygously (Table 1). All patients with the deletion of CaM KMT present severe growth retardation, severe neonatal hypotonia and moderate mental retardation. When tested, a mitochondrial deficiency was observed (12,18). All these clinical presentations can now be attributed to the absence of *CaM KMT*. The absence of the *PREPL* gene in the smallest deletion could contribute to the growth retardation and reduced muscle function as demonstrated by the mouse model deleted of *PREPL* (19). Thus, *CaM KMT* could present a new developmental delay gene, whether its effect could be all attributed through its effect on the mitochondria remains to be determined.

**Table 1:** Comparison of clinical features of different homozygous deletion in 2P21.

Name	Deleted genes	Clinical observations	References
2P21 deletion	*PP2Cβ*, *SLC3A1*, *PREPL* and *CaM KMT*	Cystinuria type I, severe growth retardation, severe neonatal hypotonia, mitochondrial defect, severe mental retardation, facial dysmorphology	12
Atypical HCS (hypotonia cystinuria)	*SLC3A1*, *PREPL* and *CaM KMT*	Cystinuria type I, severe growth retardation, severe neonatal hypotonia, Mitochondrial complex IV deficiency, mental psychomotor retardation, facial dysmorphology (different from the 2P21 deletion)	18
HCS	*SLC3A1* and *PREPL*	Cystinuria type I, moderate growth retardation, severe neonatal hypotonia,	13,16
No name	*PREPL* and *CaM KMT*	severe growth retardation, severe neonatal hypotonia, moderate mental retardation	17

The current study has clinical implications for human patients. Patients presenting slow growth, muscle weakness that could result from a mitochondrial impairment and mental retardation could have a mutation in the CaM KMT gene, should be considered for sequence analysis of the *CaM KMT* gene.

## Materials and Methods

### Ethics statement

The study was approved by the Institutional animal care and use committee of the Ben-Gurion University of the Negev. Ben-Gurion University of the Negev's animal care and use program is supervised and fully assured by the Israeli Ministry of Health; it is operated according to Israel's Animal Welfare Act 1994 and follows the Guide for Care and Use of Laboratory Animals (NRC 2011). In addition, BGU is assured by the Office of Laboratory Animal Welfare, USA (OWLA) #A5060-01, and fully accredited by the Association for Assessment and Accreditation of Laboratory Animal Care International (AAALAC). Mice were sacrificed by CO2.

### Generation of the knockout construct

The construction of the knockout construct was done in a stepwise manner. A β-gal-Neo cassette flanked by 50bp homology regions matching the boundaries of the desired deletion was constructed in pBR322 (S1A Fig). Briefly, a nuclear localization signal containing β- galactosidase gene (pNlacf, a gift from Richard Palmiter) was amplified using the following primers, Forward-CCGCGGGAGAGCCGTGGCGGACGCAACCTCCGGGTCCAGTCAGCTCTGGAGATGGGGCCCAAGAAGAA Reverse–CCGCGGCAGCATGCCTGCTATTGTCT, the forward primer contained a 50bp homologous region to the 5’ UTR of the CaMKMT. The neomycin resistance (pEGFP-N1 Clontech) gene was amplified using the following primers, Forward- ATAACTTCGTATAATGTATGCTATACGAAGTTATGCACTTTTCGGGGAAATGTG, Reverse–GGTGACCAGGCTGGGAGTATAACTTCGTATAGCATACATTATACGAAGTTATGACGCTCAGTGGAACGAAAA, both primers contained a 35bp LoxP sequence whereas the reverse primer also contained a 50bp sequence homologous to position 300–350 of the first intron of CaMKMT. The two PCR products were cloned to the pGEM-t easy vector (T-A cloning, Promega) and further subcloned into pBR322 (S1B Fig). The above construct was then used to modify a BAC containing the CaMKMT region (S1B Fig) of the mouse strain 129S7/AB2.2 (a gift from the Sanger Institute, the AB2.2 BAC library: bMQ-132H2). The modification was performed using the recombineering method that uses phage λ proteins that facilitate homologous recombination in E.coli [30]. Briefly, the BAC was transformed into DY380 bacterial strain (kindly provided by Dr Neal Copeland, NCI-Frederic, MD, USA); resistant colonies were further transformed with the β-gal-Neo cassette (excised from pBR322 by restriction digestion). Resistant colonies were analyzed by PCR for proper integration at the 5’ and 3’ sites, for each site one primer was within the cassette and the other in the homologous region in the BAC. Primers for 5': TGGGCTTTTTCTCTTCCTGA, GTTTTCCCAGTCACGACGTT (primers A and B, S1C Fig); Primers for 3': CGTGAGTTTTCGTTCCACTG, GAGCGGTTTGGAAGACTGAA (primers C and D, S1C Fig). An example of PCR products matching correct integration can be seen in S1D Fig. To generate the knockout construct that will be transfected into ES cells, the entire pBR322 was amplified by PCR using two primers which had at the 5’ and 3’ ends a 50bp sequence homologous to regions 8120bp upstream and 3446bp downstream to the β-gal gene respectively. Transformation of DY380 containing the modified BAC with the pBR322 based PCR product generated a pBR322 plasmid that contained a 8120bp homology arm upstream to the β-gal gene and 3446bp homology arm downstream to the β-gal gene. The Knockout cassette including the homology arms was cut from the pBR322 backbone by digestion with SgsI and transfected into ES cells from mouse strain W4/129SvEv at the University of Iowa Knockout core. Similarly to the primer set used to verify correct integration into the BAC, the primer sets for verification of homologous recombination in ES cells included one within the cassette and the other outside of the homology region used for recombination. Primers for the 5' integration site: GTCTTTGGAACGAACCAAGCAGCA, TGTGCTGCAAGGCGATTAAGTTGG. Primers for the 3' integration site: CAATACGCCCGCGTTTCTTCCTTT, TAATGCCAGTTCTTGGGAGGCAGA. This would create PCR products of 3878bp for the 3' side and 8857 bp for the 5' side. Out of 192 clones resistant to G418, three showed homologous recombination (S2 Fig).

PCR genotyping of the mice was done using primers: Forward: 5' CCTGACAGGGAAGAAGTTGG 3' and Reverse: 5' CGCCTCTGCCTCAGTCTCT 3' to detect the wild type genotype and primers Forward: 5' GTGCACGGCAGATACACTTG 3' Reverse: 5' GATGGCTGGTTTCCATCAGT 3' to detect the knock out sequence. PCR conditions were:30 cycles at 94° 40sec., 62° 45 sec. and 72° 1 min. followed by 10 min at 72°, using DreamTaq (ThermoScientific)

### RT-PCR

RNA was extracted from kidney using DirectZol (ZymoResearch, USA). cDNA was produced from 3 microgram RNA with Superscript II (InVitrogene). The quality of the resulting cDNA has been tested by the amplification of GAPDH with the primers: forward-5′AAAACGTCCATGTTCCCATC3′; reverse-5′CCCCAGACACGATAAGCAGT3. The primers for PCR of CaM KMT were from the first exon: 5' TGCAGAGACTGAGGCAGAGG 3' and from the second exon: 5' TGTGGCTTCTGTGACTGAGAAC 3'. PCR was performed on 1 microliter of cDNA, conditions were:40 cycles at 94° 40sec., 63° 45 sec. and 72° 1 min. followed by 10 min at 72°, using DreamTaq (ThermoSCIENTIFIC).

### Western analyses

Western analyses were performed as detailed in [11] using the polyclonal CaM KMT antibody described [11]. β-Gal activity was detected by ONPG analysis (Sigma) according to manufacturer instructions and [20].

### Histological examination

Was done by Bactochem Ltd. Israel on 2 mice at each 21 days and 9 weeks that included: kidney, pancreas, testis, intestine, lung, brain, heart, thymus, striated muscle, salivary gland, and barderian gland, lymph node. The Hematoxilin Eosin staining on the adult mice was done by the standard procedure.

### Analysis of free amino acids including Cystine in urine samples of mice

Analysis of free amino acids including Cystine in urine samples of mice was done according to Moore, S., and Stein, W. H. (1948) [31]. 100μl of the urine sample were transferred into an Eppendorf vial and 250μl of Acetonitrile were added into this vial and the solution was vortex. After centrifuge for 5 minutes at 5000rpm 300μl of the upper phase were transferred into another vial and evaporated to dryness, by a concentrating centrifuge. The dry extract was dissolved by 250μl amino acid sample buffer, vortex and filtered by using 0.45μm nylon filter. 25μl were injected into the Amino-Acid-Analyzer {AAA- Knauer model A200, Column & packing: High resolution Lithium column for AAA, 80 x 4.6 mm, Temp. 30–60°C; Mobile phase: Lithium buffers for physiological amino acid analysis (With variable pH and lithium concentrations). One of the samples was spiked with Cystine standard and the preparation and analysis was done as described above. The Cystine and other amino acids were separated on an ion exchange column and derivatized with Ninhydrin after their elution from the column (Post column derivatization). The Cystine and the amino acids were detected at 570nm and 440nm. The Cystine and the amino acids were identified and quantified against standard that was injected into the AAA. The concentration of each amino acid in the sample (mg/L) was calculated using an Excel program.

### Staining of mouse brain

Mice were perfused with cold PBS pH 7.4, brains were excised and sliced at 0.3mm intervals using a vibrotome (Leica VT1200), Brains were washed in PBS for 5 min. and fixed with cold 1.5% formaldehyde, 0.2% glutaraldehyde, 5mM EGTA and 2mM MgCl_2_ in PBS for 1 hr. Two washes were done at 4 ° followed by an overnight wash at 37° with wash buffer: 2 mM MgCl_2,_ 0.01% deoxycholate, 0.02% NP40 in PBS, before transfer to X-Gal staining buffer at 37° for 10hrs. The slices were washed 30 min. at 37° and twice again for 30 min at room temperature. The slices were embedded in glycergel containing half volume of glycerine, 5% gelatin and 1% phenol in water and kept at 4 ° until visualized.

### CaM methylation assays

Were done as detailed in [7] and [11]. Tissues and organs excised from WT or KO adult female mice were extracted in buffer 50mM Tris pH = 7.5, 150mM KCl, 2mM MgCl2, 2.5mM MnCl2, 0.01% Triton X-100, 2mM CaCl2 and 2mM TCEP. Protein content in the lysates was measured using the Bradford protein assay. One hundred ug of total proteins were incubated for 2h at 37C in presence of 5ug *Hs*CaM KMT and ^3^H-methyl Adomet in 50ul reaction containing 50mM Tris pH = 7.5, 150mM KCl, 2mM MgCl2, 2.5mM MnCl2, 0.01% Triton X-100, 2mM CaCl2 and 2mM TCEP. Samples were precipitated in 10%TCA and resuspended in 30ul blue loading dye. Samples were run a 12.5% SDS-PAGE gels that were then treated with Enhance (Perkin Elemer), dried and exposed to X-ray film. Control samples contained only 0.67ug of CaM and CaM KMT.

### CaM enrichment

Lysates from muscle tissues (both WT and KO) were loaded on a 100μl phenylsepharose 6FF column equilibrated in the presence of 5mM CaCl2. After extensive washing CaM was eluted by addition of 50mM Tris pH = 7.5 and 10mM EGTA. The whole elution was TCA precipitated, loaded on SDS-PAGE gel and the band excised and submitted for MS/MS analysis.

### Respiratory chain analysis

The enzymatic activities of respiratory chain complexes in isolated mitochondria were measured at 37°C by standard spectrophotometric methods as described [32,33]. Briefly Complex I was measured as rotenone sensitive NADH-CoQ reductase monitoring the oxidation of NADH at 340nm in the presence of coenzyme Q1. Complex I+III was measured as rotenonene sensitive NADH- cytochrome *c* reductase at 550 nm. Complex II was measured as succinate dehydrogenase (SDH) based on the succinate mediated phenazine methosulfate reduction of dichloroindophenol at 600nm. Complex II+III was measured as succinate cytochrome c reductase and following the reduction of oxidized cytochrome c at 550 nm. Complex IV (COX, cytochrome c oxidase) was measured by following the oxidation of reduced cytochrome c at 550nm.

Citrate synthase (CS), an ubiquitous mitochondrial matrix enzyme, serving as a control, was measured in the presence of acetylCoA and oxaloacetate by monitoring the liberation of CoASH coupled to 5',5'-dithiobis (2-nitrobenzoic) acid at 412nm. Activities were calculated as μmol/min/mg protein (U/mg) and presented ratio (U/U) to CS.

### Newborn mice

Behavioral analyzes: Newborn behavior was examined as follows: the mother was removed from the home cage every day during postnatal days 4–21 to examine the newborns in the cage. Newborn mice were tested in sequential order. Each newborn was tested, marked gently by non-toxic paint, wiped lightly with nesting material with maternal scent, and returned immediately to the nest.

#### General health

Newborn general health was estimated by measurement of body weight.

#### Motor and sensory reflex development

Reflex development was assessed between birth and weaning of newborns.

Tests measuring sensory-motor function, coordination and strength:

#### Righting reflex

Was measured in seconds, as the time required for a newborn placed on its back to right itself on all four limbs. "Success" in the task was defined as the day on which newborns achieved the criteria of righting in less than 3 sec [34].

#### Attraction to auditory stimulus

A plastic platform (10 cm X 15 cm) covered with a net, and protected at the edges by soft walls, so that a 3 cm track was formed. The newborn was placed at the center and auditory stimulus was given by movement of pen tip. The ability of mice to move towards the stimulus in response to the noise of gentle scratch to the paper sand was measured. The response of the mice was scored 0 in cases when the newborn did not respond to the stimulus. A score of 0.5 was given when the newborn turned his head to the direction of the sound and a score of 1 was given when the newborn moved towards the stimulus 3 times in a raw.

#### Wire hanging

Test muscle strength by measurement of the time until the newborn release his hold of a vertical wire and land on a soft material placed 2cm under the wire. The test was repeated 3 sequential times and the mean of the repeats was recorded.

#### Nest finding

The newborn was placed at a distance of 2 cm from the nest border and followed for 60 sec. The test was repeated 3 sequential times. Score 0 was given when the mouse made no attempt to return to the nest, 0.5 was the score given when the mouse arrive to the nest at least once or move in the direction towards the nest. Successful arrival to the nest 3 times in a row was scored by 1.

### Analyses of adult mice

At age 3 months, mice were tested in a battery of behavioral tests addressing autistic like behavior in the adult mice. A week before the experiments began; the mice were separated and placed in individual cages to avoid the effect of social hierarchy on mice behavior. During that week, the mice were handled daily by the experimenter for 2 min to adapt the mice to the presence of the experimenter. All experiments were videotaped and analyzed offline using “EthoVision” software (Noldus, Netherlands). All mice were tested daily between 16:00 and 20:00. In all the behavioral tests, males and females were tested in separate sessions. The testing arena was cleaned between trials with 70% ethanol.

#### Open field task

General behavior and exploration were tested in an open field arena. Mice were placed for 5 min in a circular arena 55 cm in diameter with 20-cm high walls. Both mobility and anxiety-related behaviors were examined in this test. The variables tested were mean walking distance and velocity and percentage of time in the arena during which the mice were in movement, total durations of time in the center and margin of the field, the number of entries into these areas, and rearing frequency [35].

#### Clinging to a grid

Mice were placed on a metal wire grid cover of a mouse cage. The grid was shaken gently so the mice would tighten their grip on the wires; then the grid was turned upside-down for 60 seconds, while the mice hung below the grid. The length of time the mice succeeded in holding on to the wires was measured [35].

#### Balance beam

A beam, 8 mm in diameter and 70 cm long, was horizontal and elevated. Enclosed escape boxes were placed at the ends of the beam. The mice were placed in the center of the beam. Three trials were made. The times required to reach the box, and an upright position and duration on the beam were measured [36]

#### Accelerating rotarod

Mice were placed on a rotarod apparatus (Rotarod/RS LSI Letica, Biological Instrument, Verese, Italy) accelerating from 4 to 40 rpm in 5 min. Trials began by placing the mouse on the rod and beginning rotation at 4 rpm, once the mouse maintained himself at this rotation rate the acceleration began at constant rate. Each trial ended when the mouse fell off the rod, and the speed and latency were recorded. Mice were tested for 3 trials with 50 min inter-trial interval in their home cage.

#### Passive avoidance

Measures the learning to avoid an aversive stimulus. The mice were placed on a vibrating platform 9 cm in diameter. When the mice stepped off the platform, a 5mA, 5 second, foot-shock was delivered. The mice were trained 10 runs a day with an interval of 30 min between runs. Similar procedure was performed during 3 days. The mice were placed on the platform and the time until they stepped off was recorded. When the mice stayed on the platform for 120 sec success was recorded and the mouse was removed from the platform and returned to the home cage. Conditioning was completed when the mice avoided stepping off the platform, based on this criterion, mice were determined as “learn” or “do not learn” [34]

#### Hot plate

The mice were placed on a horizontal surface that was heated to 50^0^, the times were measured until a rear paw was licked. Three trials were held at 30 minute intervals [34].

#### Olfactory test

Mice were removed from their cage and a cheese cube (1 cm2) was hidden in the cage bedding. Mice were put back in the cage, and the latency to locate the cheese was measured [34].

### Statistical analysis

Statistical analyses were performed using SPSS 18.0 software. Univariate general linear model (GLM) analysis was used to test the effects of genotype. ANOVA for repeated measurements was used for variables that were repeatedly measured at different time points. In all tests equal variance of the data not assumed therefore Dunnett T3 Post-hoc was used for multi comparisons. 2-tails student T-test was used when 2 groups were compared. In all tests differences with a *p*-value < 0.05 were regarded as significant. Results are presented as the mean ± SD.

## Supporting Information

S1 Fig Generation of the CaM KMT knockout construct. A. Schematic representation of the β-gal-Neo knockout cassette. The β-gal and a floxed Neomycin resistance gene flanked by 50bp homology regions corresponding to the 5’ UTR and the first intron of the CaM MKT locus respectively were cloned in pBR322. B. Schematic representation of the 5’ part of the CaM MKT locus, the integration sites of the knockout cassette are marked by arrows. C. The 5’ part of the CaM MKT locus following homologous recombination with the knockout cassette. Location of the primers used to detect correct integration are marked with arrows. Primers A and B for the 5' integration site and primers C and D for the 3' integration site (Primers A and D correspond to regions outside not included in the knockout cassette whereas C and D are found within the cassette). D. Results of the PCR amplifications demonstrating the correct integration of the knockout cassette into the homology arms.
(TIF)

S2 Fig Homologous recombination in G418 resistant ES cells detected by PCR. A. Scheme of the designed primers. B. and C. Correct integration at the 5’ and 3’ sites respectively was detected by PCR, in both cases one primer was outside of the knockout cassette whereas the other one was inside. The numbers above the lanes are of the clones showing homologous recombination,—no DNA.
(TIF)

S3 Fig Validation of translation in the knock out mice. Liver and kidney homogenates of CaM KMT^-/-^:-**/-**, CaM KMT^+/+^: +/+ were analyzed by Western blotting with purified CaM KMT polyclonal antibody [11]. Adjacent lanes on the same gel contained the indicated amounts of protein lysates of HEK293 transfected (+) or not transfected (-) with myc-CaM KMT pCDNA3 vector to serve as a marker for the size of CaM KMT. 60 μg of human lymphoblasts and mouse tissues lysates were separated on 12% PAA gel. Page ruler prestained protein ladder (#SM1811 Fermentas): Ma. The figure is the presentation of the full Western blot for Fig 1D.
(TIF)

S4 Fig Quantitative PCR of PRELP cDNA in quadriceps (hind limb) muscles of mice. The results represent an experiment done in triplicate of 2 C57Bl6J mice 2 months old of each genotype. The qPCR data was analyzed with the ABI 7500 Software V2.0.3 (DCt method; normalization against GAPDH). Values are expressed as RQ, means+s.d. The PRELP primers yielded a linear standard curve with an R2 0.99. The primers used were in different exons, PRELP 1510F: GCACTGTGTAATTCTAAGCCAGA, 1733R: GCTGTGATGTGAACTGAAGGATA; GAPDH F: CGACTTCAACAGCAACTCCCACTCTTCC, R: TGGGTGGTCCAGGGTTTCTTACTCCTT
(TIF)

S5 Fig Measurements of cystine in the urine of two CaM KMT^-/-^ C57Bl6J male adult (4 and 10 month old) mice in comparison to comparable CaM KMT^+/+^. The upper chromatograms presents some of the amino acid composition in Cam KMT^-/-^ C57Bl6J:KO, cysine is missing. The lower chromatograms of CaM KMT^+/+^:WT shows the same. The urine sample presented below was spiked with cystine prior the analysis to demonstrate its expected elution. The measurements were done by an Amino acid analyzer (Knauer model A200 with a high resolution Lithium column for AAA, 80 x 4.6 mm). The eluents were lithium buffers with variable pH and lithium concentrations. The cystine and other amino acids were derivatized with ninhydrin after their elution from the column and were detected at 570nm.
(TIF)

S6 Fig Fragmentation on Maldi TOF of peptides from CaM. CaM was extracted from 2 adult females CaM KMT^+/+^. The trypsin digestion was submitted for MS/MS analysis and the peptides corresponding to masses 2401Da (uncut peptide H107-R126 containing methylated K115; panel A) and 1349 Da (peptide L116-R126, product of trypsin digestion after unmethylated K115; panel B) were fragmented. A peptide of 1028Da corresponding to H107-K115 with a not methylated K115 was not visible in the spectra of the samples analyzed. A total of 11 of the possible b and y ions are indicated in the 2401Da spectrum and a total of 7 possible ions are indicated in the 1349Da spectrum. More ions were visible in a magnified image.
(TIF)

S1 Table Developmental profile test. The developmental profile of each test is expressed as the day the first mice reach criteria, the day 50% of mice reach criteria and the day 100% of mice reach criteria. CaM KMT^+/+^: WT, CaM KMT^+/-^: HET, CaM KMT^-/-^: KO.
(DOCX)

S2 Table Developmental profile statistics. ANOVA for repeated measurements, * weekly measurement evaluated. CaM KMT^+/+^: WT, CaM KMT^+/-^: HET, CaM KMT^-/-^: KO.
(DOCX)

S3 Table Adult mice behavior. CaM KMT^+/+^: WT, CaM KMT^+/-^: HET, CaM KMT^-/-^: KO
(DOCX)
