# Supplementary figures and images for: Calmodulin Methyltransferase Is Required for Growth, Muscle Strength, Somatosensory Development and Brain Function
Source: PLoS Genet. 2015 Aug 6;11(8):e1005388. doi: 10.1371/journal.pgen.1005388 (PMC4527749; doi:10.1371/journal.pgen.1005388)

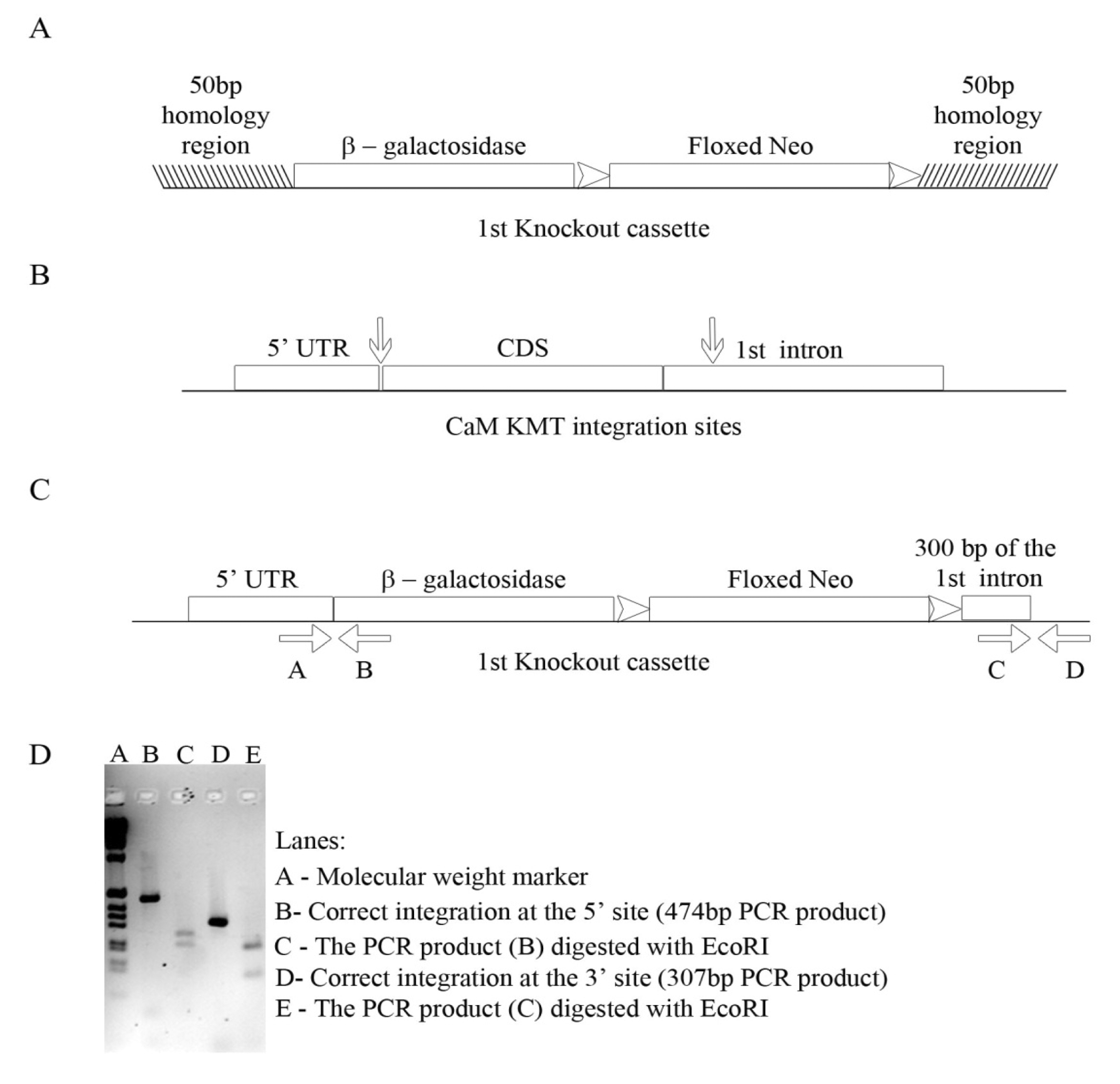

Supplement: S1 Fig — A. Schematic representation of the β-gal-Neo knockout cassette. The β-gal and a floxed Neomycin resistance gene flanked by 50bp homology regions corresponding to the 5’ UTR and the first intron of the CaM MKT locus respectively were cloned in pBR322. B. Schematic representation of the 5’ part of the CaM MKT locus, the integration sites of the knockout cassette are marked by arrows. C. The 5’ part of the CaM MKT locus following homologous recombination with the knockout cassette. Location of the primers used to detect correct integration are marked with arrows. Primers A and B for the 5' integration site and primers C and D for the 3' integration site (Primers A and D correspond to regions outside not included in the knockout cassette whereas C and D are found within the cassette). D. Results of the PCR amplifications demonstrating the correct integration of the knockout cassette into the homology arms. (TIF) [file pgen.1005388.s001.tif]

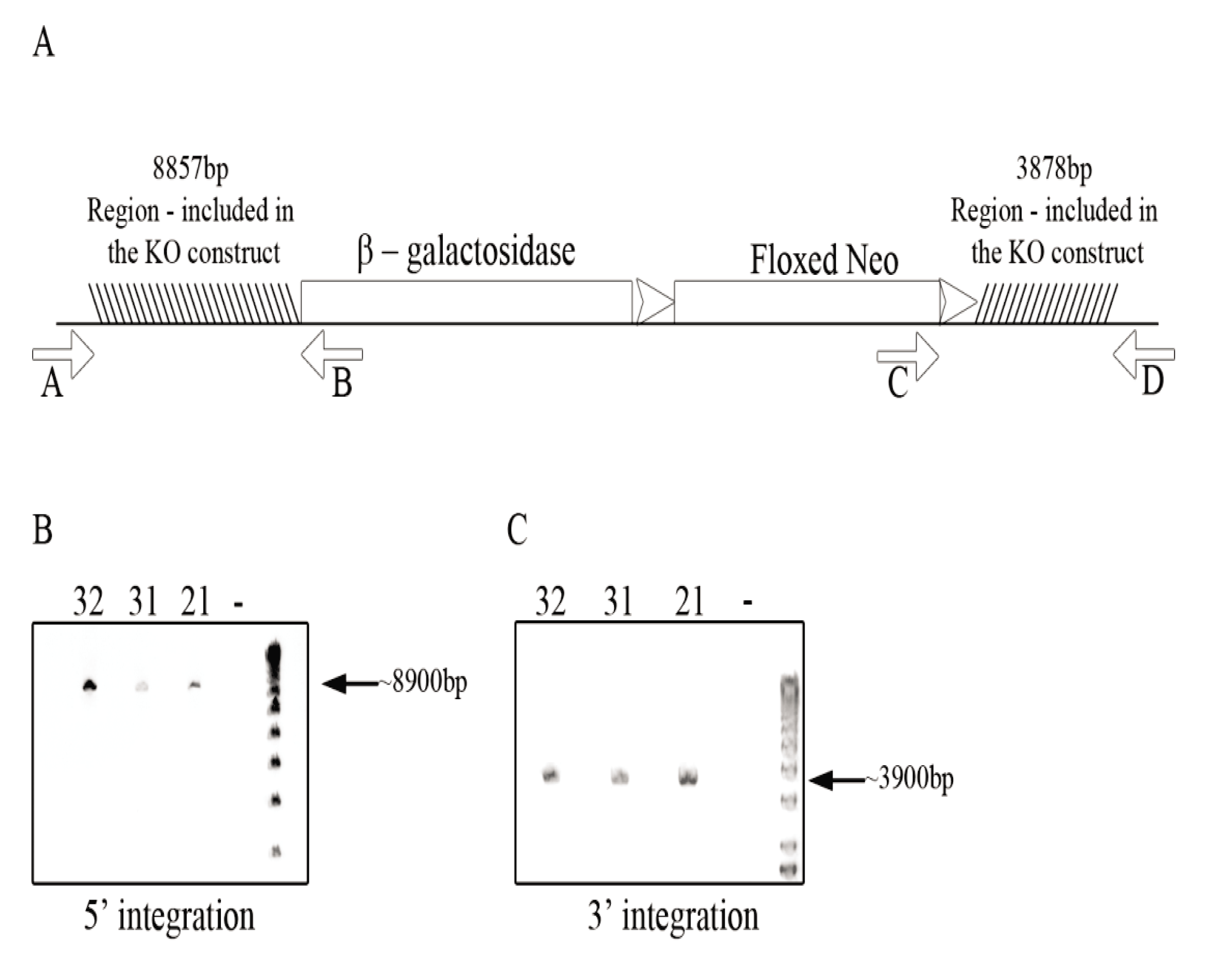

Supplement: S2 Fig — A. Scheme of the designed primers. B. and C. Correct integration at the 5’ and 3’ sites respectively was detected by PCR, in both cases one primer was outside of the knockout cassette whereas the other one was inside. The numbers above the lanes are of the clones showing homologous recombination,—no DNA. (TIF) [file pgen.1005388.s002.tif]

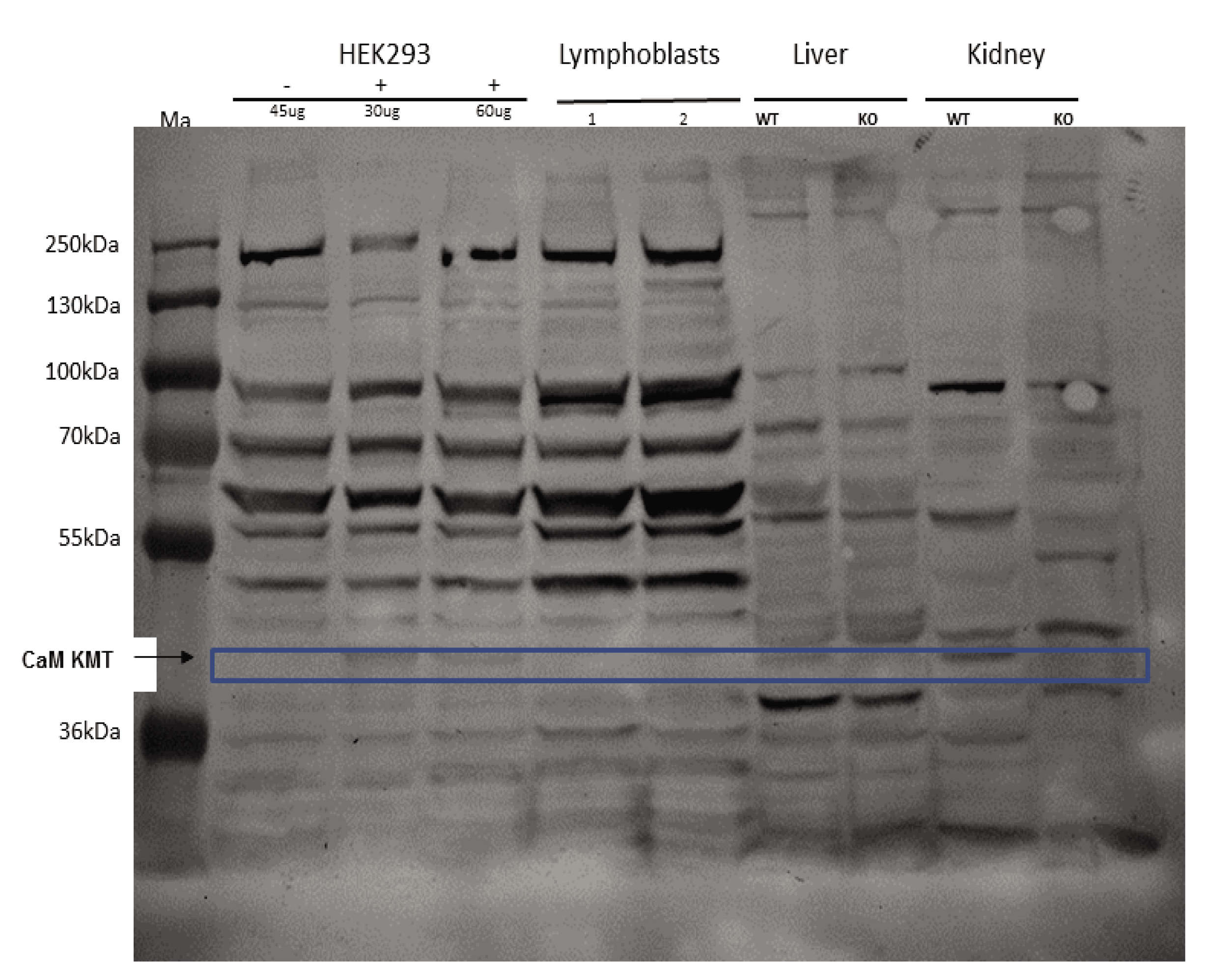

Supplement: S3 Fig — Liver and kidney homogenates of CaM KMT-/-:-/-, CaM KMT+/+: +/+ were analyzed by Western blotting with purified CaM KMT polyclonal antibody [11]. Adjacent lanes on the same gel contained the indicated amounts of protein lysates of HEK293 transfected (+) or not transfected (-) with myc-CaM KMT pCDNA3 vector to serve as a marker for the size of CaM KMT. 60 μg of human lymphoblasts and mouse tissues lysates were separated on 12% PAA gel. Page ruler prestained protein ladder (#SM1811 Fermentas): Ma. The figure is the presentation of the full Western blot for Fig 1D. (TIF) [file pgen.1005388.s003.tif]

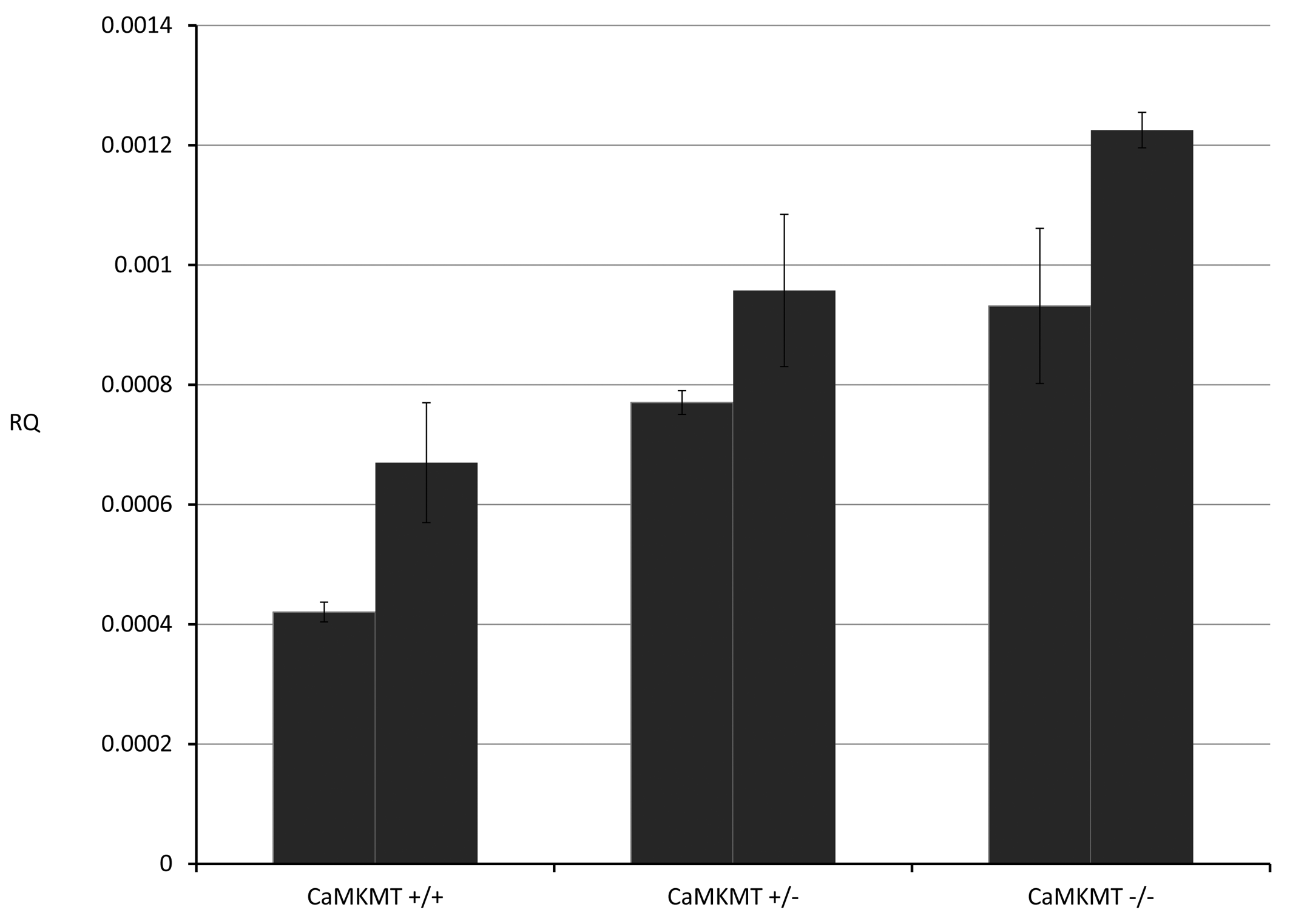

Supplement: S4 Fig — The results represent an experiment done in triplicate of 2 C57Bl6J mice 2 months old of each genotype. The qPCR data was analyzed with the ABI 7500 Software V2.0.3 (DCt method; normalization against GAPDH). Values are expressed as RQ, means+s.d. The PRELP primers yielded a linear standard curve with an R2 0.99. The primers used were in different exons, PRELP 1510F: GCACTGTGTAATTCTAAGCCAGA, 1733R: GCTGTGATGTGAACTGAAGGATA; GAPDH F: CGACTTCAACAGCAACTCCCACTCTTCC, R: TGGGTGGTCCAGGGTTTCTTACTCCTT (TIF) [file pgen.1005388.s004.tif]

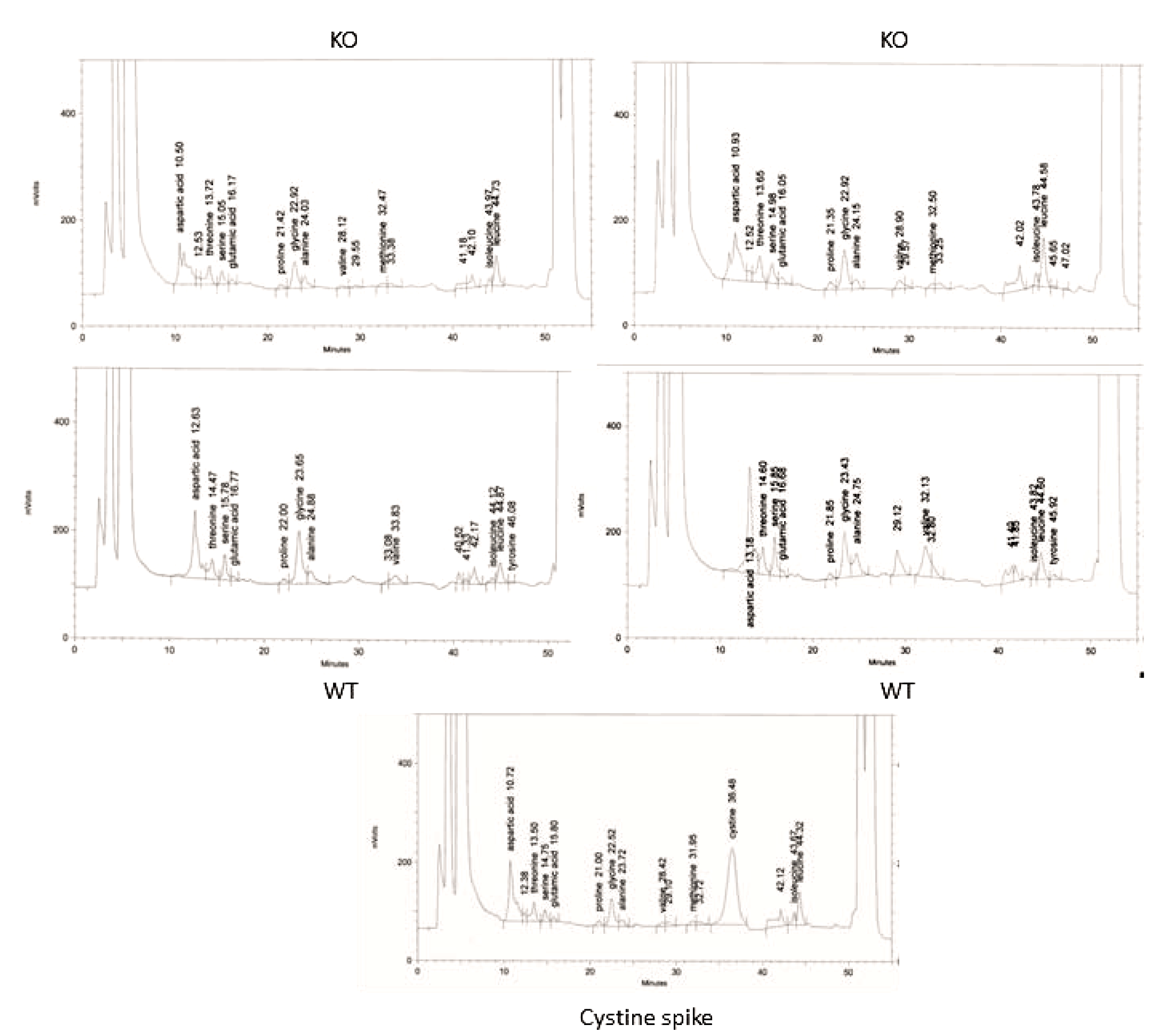

Supplement: S5 Fig — The upper chromatograms presents some of the amino acid composition in Cam KMT-/- C57Bl6J:KO, cysine is missing. The lower chromatograms of CaM KMT+/+:WT shows the same. The urine sample presented below was spiked with cystine prior the analysis to demonstrate its expected elution. The measurements were done by an Amino acid analyzer (Knauer model A200 with a high resolution Lithium column for AAA, 80 x 4.6 mm). The eluents were lithium buffers with variable pH and lithium concentrations. The cystine and other amino acids were derivatized with ninhydrin after their elution from the column and were detected at 570nm. (TIF) [file pgen.1005388.s005.tif]

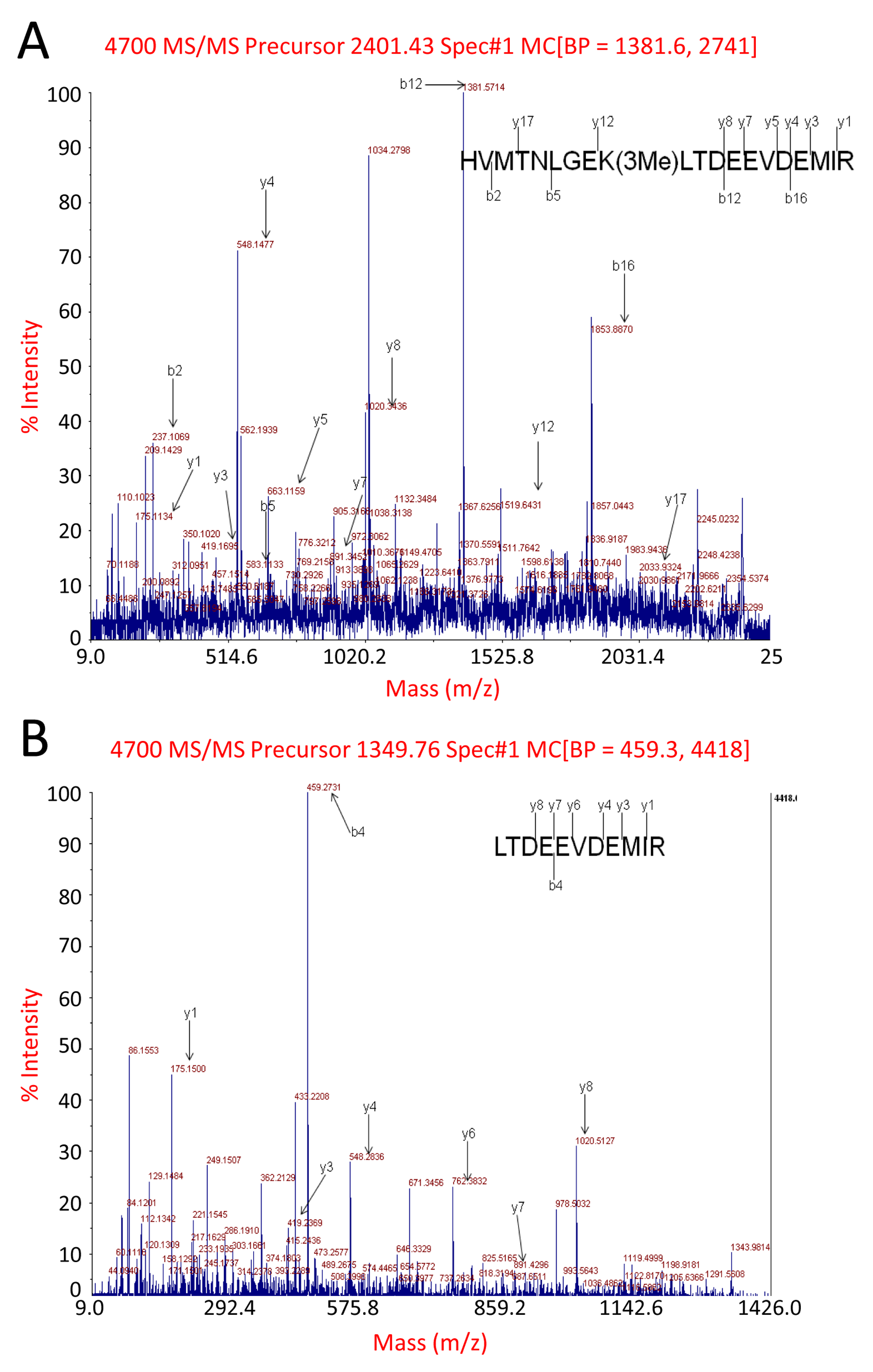

Supplement: S6 Fig — CaM was extracted from 2 adult females CaM KMT+/+. The trypsin digestion was submitted for MS/MS analysis and the peptides corresponding to masses 2401Da (uncut peptide H107-R126 containing methylated K115; panel A) and 1349 Da (peptide L116-R126, product of trypsin digestion after unmethylated K115; panel B) were fragmented. A peptide of 1028Da corresponding to H107-K115 with a not methylated K115 was not visible in the spectra of the samples analyzed. A total of 11 of the possible b and y ions are indicated in the 2401Da spectrum and a total of 7 possible ions are indicated in the 1349Da spectrum. More ions were visible in a magnified image. (TIF) [file pgen.1005388.s006.tif]
