# Supplementary material for: Calmodulin Methyltransferase Is Required for Growth, Muscle Strength, Somatosensory Development and Brain Function
Source: PLoS Genet. 2015 Aug 6;11(8):e1005388. doi: 10.1371/journal.pgen.1005388 (PMC4527749; doi:10.1371/journal.pgen.1005388)
Supplement: S1 Table — The developmental profile of each test is expressed as the day the first mice reach criteria, the day 50% of mice reach criteria and the day 100% of mice reach criteria. CaM KMT+/+: WT, CaM KMT+/-: HET, CaM KMT-/-: KO. (DOCX) [file pgen.1005388.s007.docx]

**Table S1: Developmental profile test**.

| **Test** | **Age (days)** | **Test goal** | **Geotype** | **First time of reaching the test goal (day)** | | **50% of mice met criteria (day)** | | **100% of mice met criteria (day)** | |
| --- | --- | --- | --- | --- | --- | --- | --- | --- | --- |
|  |  |  |  | *First time mice met criteria (day)* | *male* | *female* | *male* | *females* | *males* |
| **Muscle strength** | 5-21 | Hanging on a wire for 60 sec | WT | 15 | 18 | 20 | 20 | - | - |
|  |  |  | HET | 17 | 17 | 21 | 21 | - | - |
|  |  |  | KO | 21 |  | - | - | - | - |
| **Nest finding** | 5-14 | Score 1 (finding nest 3 times) | WT | 8 | 8 | 10 | 10 | 13 | 13 |
|  |  |  | HET | 8 | 8 | 10 | 10 | 13 | 13 |
|  |  |  | KO | 8 | 9 | 12 | 11 | 13 | 14 |
| **Sensory attraction** | 5-15 | score 1 (moving toward vibration source 3 times) | WT | 10 | 10 | 12 | 12 | 14 | 14 |
|  |  |  | HET | 10 | 9 | 12 | 12 | 14 | 14 |
|  |  |  | KO | 10 | 12 | 13 | 14 | 14 | 15 |

The developmental profile of each test is expressed as the day the first mice reach criteria, the day 50% of mice reach criteria and the day 100% of mice reach criteria. CaM KMT^+/+^ : WT, CaM KMT^+/-^ : HET , CaM KMT^-/-^ : KO.
