# Supplementary material for: Calmodulin Methyltransferase Is Required for Growth, Muscle Strength, Somatosensory Development and Brain Function
Source: PLoS Genet. 2015 Aug 6;11(8):e1005388. doi: 10.1371/journal.pgen.1005388 (PMC4527749; doi:10.1371/journal.pgen.1005388)
Supplement: S2 Table — ANOVA for repeated measurements, * weekly measurement evaluated. CaM KMT+/+: WT, CaM KMT+/-: HET, CaM KMT-/-: KO. (DOCX) [file pgen.1005388.s008.docx]

**Table S2: Developmental profile statistics.**

| ***Repeated Measure ANOVA*** | | | | | | | | | | | | |
| --- | --- | --- | --- | --- | --- | --- | --- | --- | --- | --- | --- | --- |
| **Test** | **Days of difference between genotypes** | | **df** | **n** | | **F** | | **P** | | **Post Hoc tests** | | |
|  |  |  |  |  |  |  |  |  |  | **(Dunnett's T3)** | | |
|  | **female** | **male** |  | **female** | **male** | **female** | **male** | **female** | **male** | **Genotype** | **female** | **male** |
| **Weight Daily** | 5-21 | 5-21 | 2 | 66 | 50 | 10.22 | 7.05 | <0.001 | 0.002 | KO *vs* WT | <0.001 | 0.138 |
|  |  |  |  |  |  |  |  |  |  | KO *vs* Het | 0.001 | 0.052 |
| **Weight Weekly** | 3-6* | 3-12* | 2 | 65 | 50 | 12.72 | 19.24 | <0.001 | <0.001 | KO *vs* WT | <0.001 | 0.003 |
|  |  |  |  |  |  |  |  |  |  | KO *vs* Het | <0.001 | 0.003 |
| **Muscle Strength** | 7-21 | 15-21 | 2 | 66 | 50 | 13.05 | 5.62 | <0.001 | 0.006 | KO *vs* WT | <0.001 | 0.07 |
|  |  |  |  |  |  |  |  |  |  | KO *vs* Het | <0.001 | 0.003 |
| **Nest finding** | 7-10 | 7-13 | 2 | 102 | 83 | 4.18 | 6.35 | 0.018 | 0.003 | KO *vs* WT | 0.016 | 0.033 |
|  |  |  |  |  |  |  |  |  |  | KO *vs* Het | 0.191 | 0.011 |
| **Sensory Attraction** | 10-13 | 12-14 | 2 | 102 | 83 | 7.56 | 10.24 | 0.001 | <0.001 | KO *vs* WT | 0.011 | 0.122 |
|  |  |  |  |  |  |  |  |  |  | KO *vs* Het | 0.137 | 0.026 |

ANOVA for repeated measurements, * weekly measurement evaluated. CaM KMT^+/+^ : WT, CaM KMT^+/-^ : HET , CaM KMT^-/-^ : KO.
