# Supplementary material for: Calmodulin Methyltransferase Is Required for Growth, Muscle Strength, Somatosensory Development and Brain Function
Source: PLoS Genet. 2015 Aug 6;11(8):e1005388. doi: 10.1371/journal.pgen.1005388 (PMC4527749; doi:10.1371/journal.pgen.1005388)
Supplement: S3 Table — CaM KMT+/+: WT, CaM KMT+/-: HET, CaM KMT-/-: KO (DOCX) [file pgen.1005388.s009.docx]

**Table S3: Adult mice behavior.**

| **Test** | **Statistic test** | **test goal** | **Genotypes compared** | **Statistic parameter** | **P** |
| --- | --- | --- | --- | --- | --- |
| **Hanging on a Grid** | t-test (between groups) | Hanging time | WT+HET *vs* KO | t=2.31 | *P=0.034* |
| **Balance Beam** | Repeated Measure ANOVA (within groups) | Time on beam | WT+HET | F(2,22)=7.88 | *P=0.003* |
|  |  |  | KO | F(2,10)=4.29 | *P=0.045* |
|  |  | Time to reach box | WT+HET | F(2,19)=6.69 | *P=0.006* |
|  |  |  | KO | F(2,7)=2.74 | P=0.132 |
| **Rota-Rod** | Repeated Measure ANOVA (between groups) | Time on rod | WT+HET *vs* KO | F(1,35)=9.48 | *P=0.004* |
|  |  | Speed at fall | WT+HET *vs* KO | F(1,35)=9.40 | *P=0.004* |
|  | Repeated Measure ANOVA (within groups) | Time on rod | WT+HET | F(2,22)=6.81 | *P=0.005* |
|  |  |  | KO | F(2,10)=1.16 | P=0.325 |
|  |  | Speed at fall | WT+HET | F(2,22)=6.09 | *P=0.008* |
|  |  |  | KO | F(2,10)=1.32 | P=0.308 |
| **Passive Avoidance** | Repeated Measure ANOVA (within groups) | staying on platform for 120 sec | WT+HET | F(2,13)=6.88 | *P=0.009* |
|  |  |  | KO | F(2,7)=1.37 | P=0.313 |

CaM KMT^+/+^ : WT, CaM KMT^+/-^ : HET , CaM KMT^-/-^ : KO
